# Characterisation of pelagic seascapes through micronektonic and zooplanktonic scattering layers

**DOI:** 10.1038/s41598-026-36104-1

**Published:** 2026-01-23

**Authors:** Ndague Diogoul, Patrice Brehmer, Julien Jouanno, Yannick Perrot, Alexandra Rosa, Jesus Reis, Cláudio Cardoso, Birgit Quack

**Affiliations:** 1 Senegalese Institute for Agricultural Research (ISRA), Centre for Oceanographic Research of Dakar-Thiaroye (CRODT), Dakar, Senegal; 2 French Research Institute for Development (IRD), Université de Bretagne Occidentale (UBO),French National Centre for Scientific Research (CNRS), French Research Institute for Exploitation of the Sea (Ifremer), Sub-Regional Fisheries Commission (SRFC), Dakar, Senegal; 3 Laboratory of Geophysics and Space Oceanography (LEGOS), University of Toulouse, French Research Institute for Development (IRD), French National Centre for Scientific Research (CNRS), French National Centre for Space Studies (CNES), Paul Sabatier University (UPS), Toulouse, France; 4 Institut de Recherche pour le Développement (IRD), Centre National de la Recherche Scientifique (CNRS), Institut Français de Recherche pour l’Exploitation de la Mer (Ifremer), Délégation Régionale Ouest, Plouzané, France; 5 Observatório Oceânico da Madeira, Edifício Madeira Tecnopolo, Funchal, Portugal; 6 Geomar Helmholtz Centre for Ocean Research, Kiel, Germany

**Keywords:** Pelagic seascape, Diel vertical migration, Micronekton, Cross-Atlantic, Ecology, Ocean sciences

## Abstract

Landscape ecology is a key discipline for studying the relationships between spatial patterns and ecological processes, as well as for monitoring macro-scale changes in ecosystems. Unlike terrestrial landscapes, which have been extensively studied, the open-ocean pelagic environment presents unique complexities that require innovative approaches for its understanding. Aggregations of micronektonic and macrozooplanktonic organisms in layers are prominent features of the open-ocean pelagic zone. Such visually cryptic features can be revealed by echosounders as pelagic Sound Scattering Layers (SSLs). We characterise various pelagic seascapes and their relationships with environmental parameters across three oceanographically contrasting tropical regions, characterised by diverse ecological patterns, using an integrated methodological framework that combines dual-frequency acoustic analysis (18 and 38 kHz). Diel vertical migration is a common feature that involves epipelagic and mesopelagic SSLs. Nevertheless, there are significant regional contrasts in SSL spatial distribution as oceanographic features influence SSL patterns and micronektonic acoustic backscatter. Acoustically defined pelagic seascapes reveal biological-physical coupling and SSL responses to oceanographic variability at meso- and macro-scales. SSL distribution was significantly driven by oceanographic variables such as temperature, chlorophyll a, salinity, oxygen, and PAR, as well as by mesoscale eddies that structured their spatial patterns, with anticyclonic eddies concentrating SSLs’ acoustic backscatter at their centres and cyclonic eddies exhibiting scattered acoustic backscatter at their peripheries. This framework enhances our ability to assess how climate variability and changing ocean conditions influence open-ocean pelagic ecosystems. Developed and demonstrated at a broad regional scale, this validated approach establishes a transferable framework for characterising pelagic habitats through integrated SSL structures, enabling its application across wider spatial and temporal domains to advance understanding of global biophysical and ecosystem dynamics.

**Supplementary Information:**

The online version contains supplementary material available at 10.1038/s41598-026-36104-1.

## Introduction

Ecological studies have extensively described and utilised terrestrial landscapes^1^, providing insights into how species and populations respond to habitat changes^2^. In contrast, seascapes have received less attention^3^, with most research focusing exclusively on benthic environments^4^ and few studies on pelagic landscapes^5,6^. The pelagic realm, encompassing the vast open waters of the world’s oceans, represents one of Earth’s largest and most dynamic ecosystems^7^. The marine pelagic ecosystem classification approach often fails to capture the ocean’s inherently dynamic nature, struggling to represent the frequent changes occurring across time and space in pelagic environments. Unlike terrestrial landscapes, which migrate slowly over time, pelagic seascapes are embedded in a turbulent, advective ocean, making them difficult to comprehend without geographical references^8^. While discrete habitats, such as those formed by planktonic assemblages, do exist within these expansive oceanic regions, the absence of clearly delineated boundaries and the inherent turbulence of marine systems complicate the perception of a stable seascape structure^3,9^. Studies have sought to delineate these dynamic pelagic structures by developing innovative approaches that integrate multiple oceanographic parameters to identify ecologically significant boundaries. These efforts range from satellite detection of oceanographic fronts and biogeochemical boundaries^8,10^ to sophisticated Lagrangian methods that identify ecological transition zones through fluid dynamics analysis^11,12^. Other studies of marine predators have revealed how mesoscale features create foraging boundaries and aggregation sites^13,14^.

The concept of “seascapes” has emerged as a powerful framework for understanding the ocean’s variable nature. Seascapes can be viewed as a mosaic of distinct habitats, each characterised by unique combinations of biological, chemical, geological, and physical processes that evolve^3,8,9^. Within this evolving conceptual framework, Sound Scattering Layers (SSLs) have emerged as a key element of pelagic seascapes. SSL are ubiquitous in all oceans^15^ and the result of acoustic scattering from extensive aggregations of micronekton and large zooplankton (to simplify hereafter only micronektonic). SSLs appear on the echogram from a scientific echosounder^16^, are vertically narrow, ranging from tens to hundreds of meters, and horizontally extensive, spanning tens to thousands of kilometres^17^. Micronekton and zooplankton species provide the main link between primary producers and higher trophic levels in the marine food web. The micronekton serves as prey for marine predators, including tunas^18^, seabirds^19^, and mammals^20^. Given their distribution in all oceans and their important biomass, mesopelagic species represent potential new fishery resources^21^.

Many marine environmental factors influence the behaviour and life history of pelagic micronekton, contributing to the spatial and temporal characteristics of SSLs^22^. Most micronektonic organisms travel vertically for hundreds of meters in the open ocean, exhibiting the diel vertical migration (DVM). They migrate from the mesopelagic domain, the part of the ocean between 200 and 1000 m depth characterised by low light levels and a distinct community of organisms adapted to these conditions, where they reside during daytime, to the epipelagic or euphotic zone, the uppermost layer of the ocean extending from the surface to a depth of about 200 m, where sufficient light penetrates to support photosynthesis, during nighttime^23,24^. The prevailing migration type is Type I, which involves ascending during nighttime to enhance feeding opportunities and descending during daytime to evade visual predators. Some planktonic and micronektonic organisms have been documented to exhibit a reverse DVM, called “Type II”, in which they ascend in the morning and descend in the evening or early night^25^ due to, e.g., specific hydrographic features, tides, food availability, or predation avoidance. DVM behaviors are influenced by environmental cues (e.g., dissolved oxygen, light, nutrients, and temperature) and predator–prey interactions^26,27^. Therefore, micronekton is a major player in carbon export and sequestration due to the DVM they carry out on a daily cycle^28,29^. Thus, DVMs represent an essential biological process in the ocean, regulating the biological carbon pump^29^. This active vertical movement of organisms and the associated diel metabolic activity (surface feeding at night and deep metabolism during the day) play a key role in regulating the carbon cycle^23^. On a global scale, sea surface temperature, primary productivity, and dissolved oxygen emerge as the principal driving factors of SSL’s vertical distribution^22,23,30^. At regional scale, the presence of oxygen minimum zones (OMZs), which are areas of low oxygen concentration typically found at mesopelagic depths, can profoundly impact the vertical distribution and behavior of zooplankton and micronekton. OMZs strongly influence environmental conditions and ecosystem structure within specific ocean biogeographical regions^31^. Organisms migrate vertically to shallower depths with higher oxygen concentrations^23^. Mesoscale oceanographic features such as eddies and frontal boundaries are important in steering the dynamics of SSLs^32^. At a finer scale, oceanographic parameters, including primary productivity^33^, dissolved oxygen levels^22,34^, light intensity^35^, temperature^36,37^, and oceanographic processes such as wind-induced mixing^38^, along with biological interactions like predation pressure^36^, create local variabilities in SSL patterns.

Describing large-scale patterns and explaining the processes that drive them becomes increasingly relevant as marine stressors increase. Large-scale studies offer a valuable opportunity to investigate the impact of macroscale oceanography on the horizontal and vertical distribution of micronekton, and consequently, the responses of organisms in the SSLs to environmental variability.

We propose that the ubiquitous, consistent horizontal and vertical patterns, as well as the environmentally driven dynamics of SSLs across the ocean, represent key characteristics that define them as elements of a pelagic seascape. This proposed pelagic seascape comprises dynamic SSL structures shaped by the interplay between organismal behavior and physical ocean processes^5^. Our study aims to characterize the variations in SSL properties and their relationships with environmental parameters across contrasted oceanographic regions characterized by diverse oceanographic features. We investigate the primary environmental factors driving the distribution and spatial variability of SSLs within this oceanographic context. Our study adopts an innovative bi-frequency methodological approach that uses different SSL descriptors first to define distinct pelagic seascapes. Each seascape, defined using SSL descriptors, represents a unique expression biological-physical coupling and thus provides an effective description of pelagic seascape structure. By exploring the biological-physical coupling across a broad geographic range, we gain deeper insights into how SSLs respond to diverse oceanographic processes and water mass characteristics, setting the stage for predicting ecosystem shifts under changing ocean conditions.

## Results

Results are presented following the three-step analytical framework described above, encompassing pelagic seascape classification, vertical structure and diel migration patterns, and environmental driver modelling.

### Clustering of sound scattering layers descriptors

**Fig. 1:**
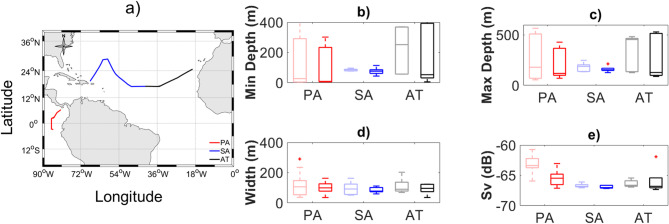
K-means clustering of (18 kHz (light colors) and 38 kHz (bold colors) acoustic data allows discrimination of three areas from 20 to 90°W (Fig. 1). (**a**) Map of the survey track with each day colored by its resulting cluster: Eastern Tropical Pacific Ocean (red; PA), Sargasso Sea (blue; SA), and Eastern Tropical North Atlantic Ocean (black; AT). (**b**–**e**) Boxplots (minimum, maximum, median, and outlier) of Sound Scattering Layer (SSLs) metrics for each identified cluster: (**b**) SSL minimal depth (m), (**c**) maximal depth (m), (**d**) width (m) and (**e**) S_v_ (dB) micronektonic acoustic backscatter.

The clustering performed on SSL acoustic descriptors (minimum depth (m), maximal depth (m), width (m), and mean micronektonic acoustic backscatter (S_v_, dB)) resulted in three clusters spatially distributed across the surveyed area (Fig. 1a). These clusters corresponded to three regions with different SSL features (Fig. 1b–e): Eastern Tropical North Atlantic Ocean (AT), Sargasso Sea (SA) and Eastern Tropical Pacific Ocean (PA). The PA cluster displayed the shallowest SSLs (~ 10 m) and the thickest SSLs (~ 150 m at 18 kHz and ~ 120 m at 38 kHz), associated with the highest micronektonic acoustic backscatter (− 62 to − 65 dB, particularly at 18 and 38 kHz). The SA region showed the thinnest SSLs (~ 115 m at 18 kHz and ~ 100 m at 38 kHz), and the lowest micronektonic backscatter (~ − 67 dB at both frequencies). The AT region displayed the deepest SSLs (~ 400 m at both frequencies).

### Sound scattering layer vertical spatial distribution

**Fig. 2:**
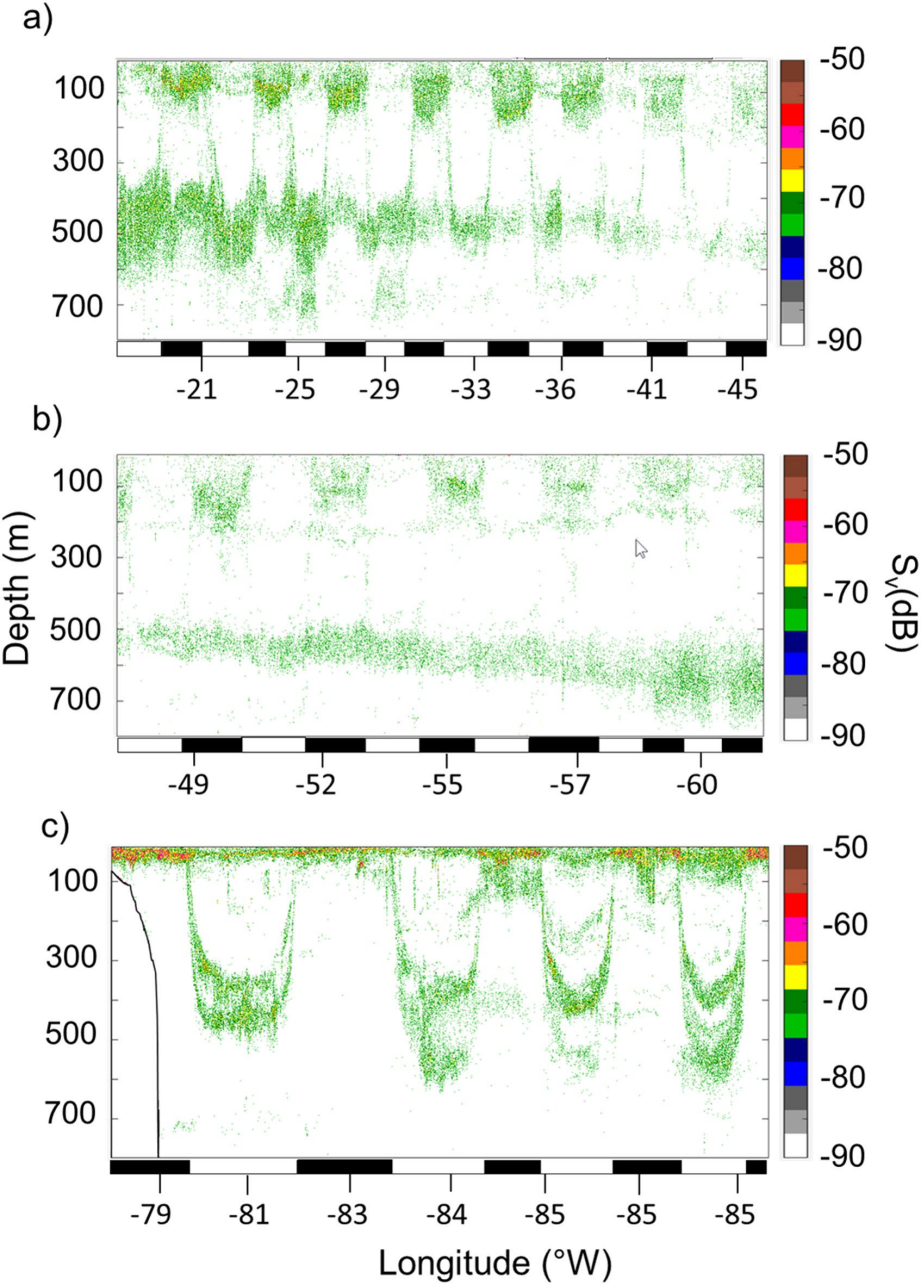
Echograms of micronektonic acoustic backscatter (S_v_ in dB) at 38 kHz illustrating their diel vertical migration in three tropical regions. (**a**) Eastern Tropical North Atlantic Ocean (AT), (**b**) Sargasso Sea (SA), and (**c**) Eastern Tropical Pacific Ocean (PA). The white and black rectangle below the echogram shows day and night periods. Acoustic detection was performed using a 38 kHz echosounder; data extraction was conducted at a − 70 dB threshold.

**Fig. 3:**
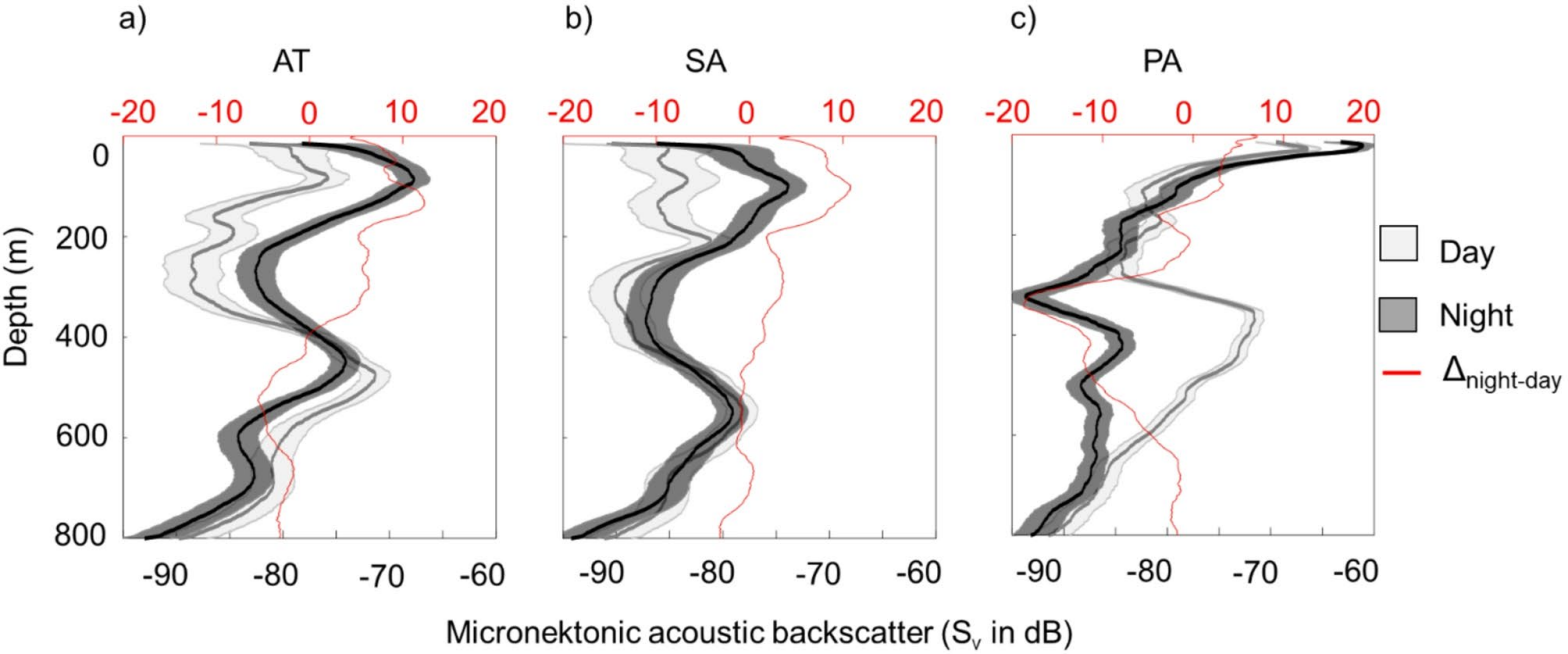
Daytime (grey) and nighttime (dark) mean vertical profile of micronektonic acoustic backscatter (S_v_ in dB) at 38 kHz within the water column in three intertropical regions. The difference between day and night ∆_night-day_ (red line and x-axis) illustrates diel vertical migration. (**a**) the Eastern Tropical North Atlantic Ocean (AT), (**b**) Sargasso Sea (SA) and (**c**) Eastern Tropical Pacific Ocean (PA). The shaded areas represent the standard deviation.

The vertical structuration of the SSLs at 38 kHz (Fig. 2) is shown for the different regions identified through SSL clustering. In the three clusters (i.e. oceanic areas), two main SSLs were observed: epipelagic SSLs between 10 and 200 m depth and mesopelagic SSLs between 300 and 700 m depth. During the day, the mesopelagic SSL resides in deeper waters, whereas at night, it migrates to shallower depths (10–150 m) to form an epipelagic SSL. The PA observed an intermediate SSL between 200 and 500 m depth. The PA region exhibited a singular feature, characterized by a continuous epipelagic SSL throughout both day and night. The vertical profile of micronektonic acoustic backscatter (Fig. 3) exhibited diverse patterns from the surface to 800 m. In the AT and PA, the diel variation in the SSL vertical distribution consistently showed higher micronektonic acoustic backscatter values in upper layers (< 400–410 m) during night-time compared to daytime, while in the deeper zone (400–800 m), the micronektonic acoustic backscatter values were higher during daytime than night-time (Fig. 3). In the PA, the DVM variation in the SSL distribution was characterized by minimal diurnal differences in the epipelagic zone (< 200 m) and the most significant differences in the mesopelagic zone (> 200 m), where daytime values exceeded nighttime values. This day-night SSL distribution pattern in the PA corresponds to the persistent presence of the surface layer regardless of the time of day, resulting in minimal diel differences in epipelagic densities. In contrast, differences are more pronounced in the mesopelagic zone. 18 kHz frequency exhibited SSLs vertical structuring (Supplementary Fig. S1) and DVM patterns (Supplementary Fig. S2) similar to those observed at 38 kHz in all three regions.

### Variability of oceanographic conditions per clustered region

**Fig. 4:**
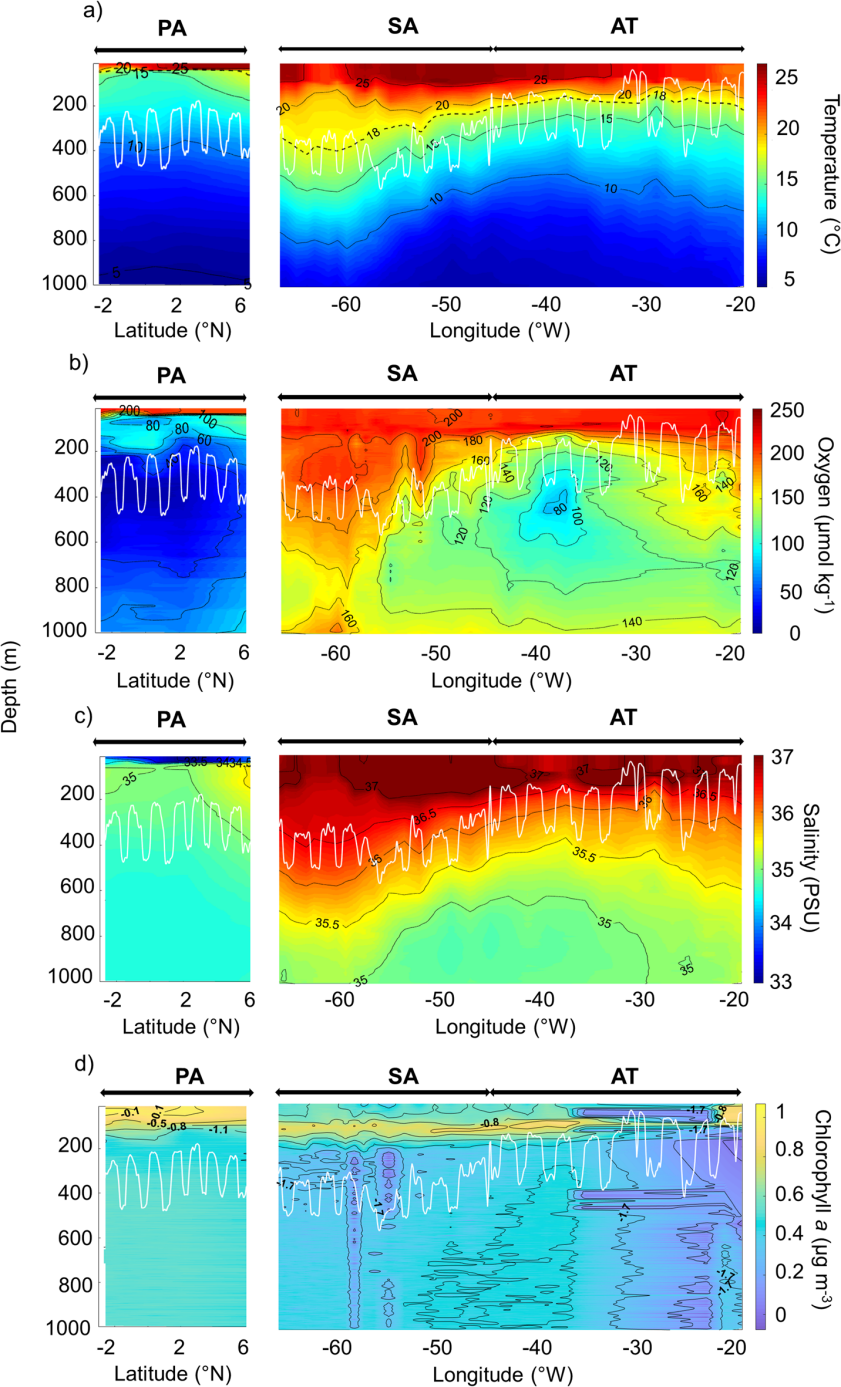
Contour plots of (**a**) water temperature with the black dotted line representing the thermocline, (**b**) dissolved oxygen, (**c**) salinity and (**d**) chlorophyll-*a* concentrations (logarithmic scale) from − 20 to − 90°W, i.e., Eastern Tropical North Atlantic Ocean (AT), Sargasso Sea (SA) and Eastern tropical Pacific Ocean (PA). White lines depict the Weighted Mean Depth (WMD, a proxy for the depth of the greatest acoustic density) at 38 kHz on each panel.

We observed warm surface waters (20–25 °C) and cold sub-thermocline waters (5–15 °C) across all surveyed regions (Fig. 4a). In the SA, the thermocline depth reaches 300 m, whereas in the AT it typically ranges from 90 to 110 m. In contrast, the PA exhibited a shallower thermocline at around 50 m depth.

The AT and SA surface waters were saturated in dissolved oxygen (> 200 μmol kg^−1^; Fig. 4b) in the upper 100 m. A relatively shallow OMZ emerged in the AT just below the productive upper strata (around 180 m depth), where dissolved oxygen concentrations reached ~ 90 μmol kg^−1^ close to hypoxia. In the SA, high oxygen concentrations (~ 200 µmol kg^−1^) also reached deep waters, i.e., 1000 m. The PA exhibited a shallower OMZ from a depth of 200 m, characterized by low dissolved oxygen concentrations, i.e., hypoxic conditions (reaching 20 μmol kg^−1^).

Surface salinity varied between 34 and 37 PSU, with higher values primarily found in the AT and SA within the upper 200 m (Fig. 4c). The PA region displayed a lower salinity of 32 PSU in the surface water above a depth of 50 m. The freshwater layer, persisting within the uppermost 50 m water column in the PA leads to a shallow halocline. In the AT and SA, the halocline is much more diffuse. Remarkably, high salinities extend to greater depths in SA, where salinities above 36.5 PSU were detected at approximately 300 m below the surface.

High chlorophyll-*a* concentrations (~ 0.5 µg m^−3^) were observed at the surface (depth < 100 m) in the eastern part of the AT (Fig. 4d). However, with increasing distance from the Canary Islands, a transition to more oligotrophic conditions was observed, as illustrated by declining surface chlorophyll-*a* concentrations that reach low values in the SA. The PA regions exhibited the highest chlorophyll-*a* concentrations, ranging from 0.4 to 1.5 µg m^−3^, in the euphotic zone (depth < 100 m).

**Fig. 5:**
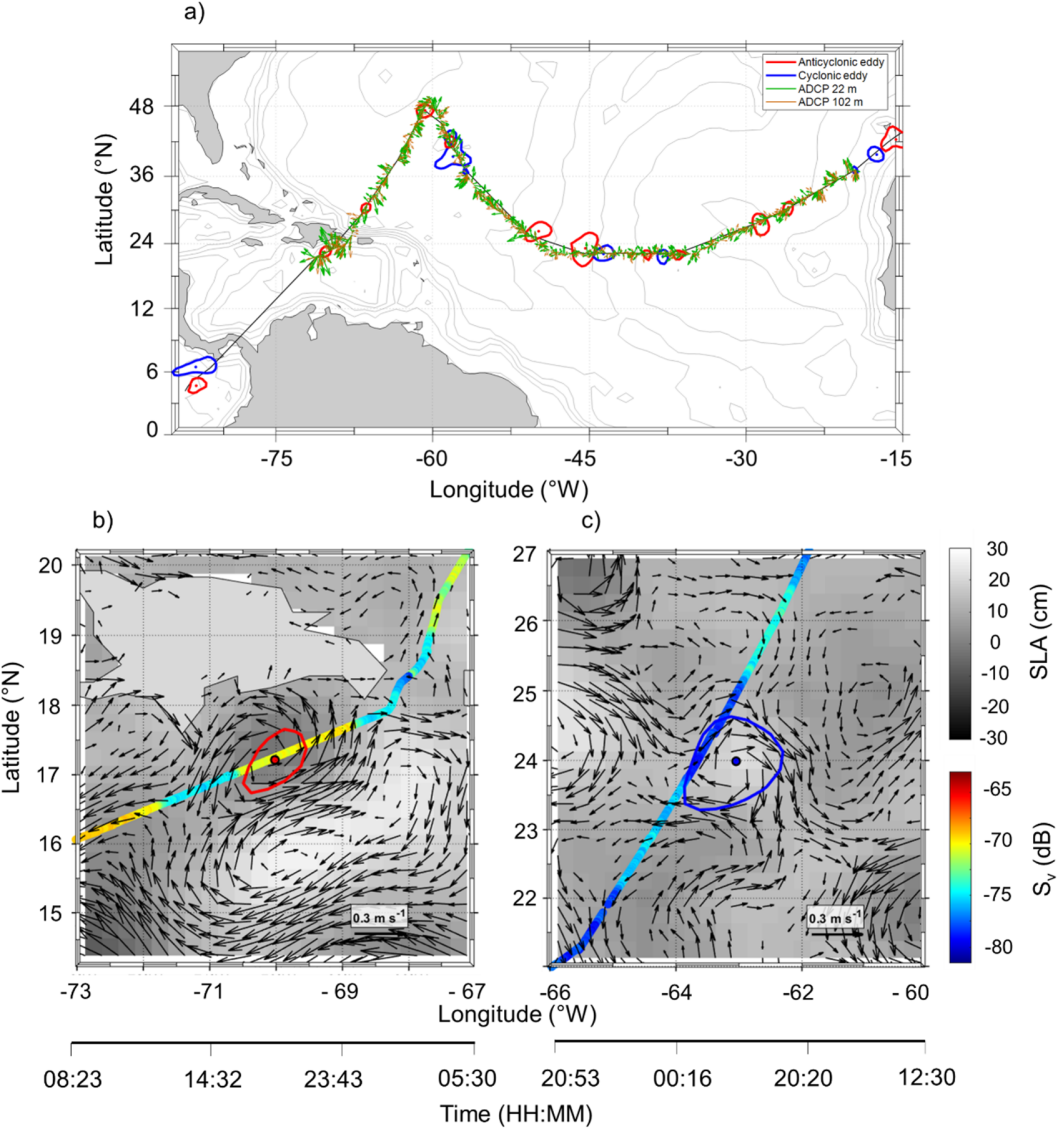
(**a**) Cruise transect showing the identification of the most relevant cyclonic (blue) and anticyclonic (red) eddies with their position, shape and trajectory. Green and brown arrows represent ocean currents at 22 m and 102 m depth, respectively, measured by the onboard ADCP (75 kHz) and averaged over 30-min intervals. Eddy-scale analysis showing, (**b**) an anticyclonic structure and (**c**) a cyclonic structure, overlaid on the mean volume backscatter (S_v_; 38 kHz, 10–200 m) and Sea Level Anomaly (SLA; grey scale). The black arrows indicate the surface geostrophic velocity derived from SLA gradients. The bottom axis depicts the time.

The cruise pathway intersects several distinct mesoscale features across the Atlantic and Pacific Oceans (Fig. 5a). In the AT, the cruise pathway encountered cyclonic eddies near the West African coast and several anticyclonic eddies further offshore. In the SA region, several anticyclonic eddies were identified along with a few cyclonic eddies. The geostrophic currents show complex patterns with moderate to strong velocities associated with these eddy structures. In the PA, anticyclonic eddies were observed as well as a large cyclonic eddy. Two dynamically distinct eddies illustrate eddy-scale acoustic patterns (Fig. 5b). Anticyclonic eddy (Fig. 5c) exhibited elevated mean S_v_ values (− 68 to − 70 dB) concentrated near its center, whereas cyclonic eddy (Fig. 5c) shows reduced backscatter in the interior (− 78 to − 80 dB) but enhanced S_v_ along the periphery (− 73 to − 75 dB).

### Effect of environmental observations on micronektonic acoustic backscatter

The GAM models revealed significant effects of the predictor variables on SSL S_v_ at 18 and 38 kHz, explaining 90.7% and 92% of the deviance, respectively (Table 1). A non-linear effect of environmental variables on micronektonic acoustic backscatter was observed (Supplementary Figs. S3, S4). For S_v18_, the most influential predictors were temperature (*p* < 0.001), followed by chlorophyll-a (*p* < 0.001), salinity (*p* < 0.001), oxygen (*p* < 0.001), and PAR (*p* < 0.001). The effect of location was also significant, confirming the spatial variability of acoustic backscatter intensity.

**Table 1:** Result of generalized additive models (GAM) depicting the relationships between micronektonic acoustic backscatter (mean acoustic volume backscattering strength S_v_ in dB) of SSLs at 18 and 38 kHz, in conjunction with environmental parameters (sea temperature, salinity, dissolved oxygen, Chlorophyll-*a* concentration, Photosynthetic Active Radiation) and location (area Eastern Tropical Atlantic Ocean, Sargasso Sea and Eastern tropical Pacific Ocean).

Variables	Significance (*p*-value)		Total deviance (%)	
	S_v18_	S_v38_	S_v18_	S_v38_
Location	0.0477*	0.0123*	90.7	92
Temperature	4.97e−11***	0.0567		
Salinity	0.0004***	3.71e−05***		
Oxygen	0.0004**	0.0119*		
Chlorophyll-a	3.8e−6***	2e−16***		
PAR	0.002**	0.0019**		

Significant values (*p*-value ≤ 0.05 marked *, *p*-value ≤ 0.01 marked **, *p*-value ≤ 0.001 marked ***

For S_v38_, chlorophyll- *a* had the most decisive influence (F = 36.8, *p* < 0.001), followed by salinity (*p* < 0.001), PAR (*p* < 0.001), and oxygen (*p* < 0.001). Temperature had a weaker but nearly significant effect (*p* = 0.056), while location remained significant (*p* < 0.001).

Temperature, chlorophyll-*a*, dissolved oxygen, PAR, and salinity were identified as key critical factors influencing the vertical distribution of SSL and backscatter intensity (Table 1), with notable effects from the thermocline and oxycline. However, it is important to note that these environmental factors, while shaping the vertical distribution, do not impose strict limitations on the vertical extent of SSLs. The observed variability in WMD (Supplementary Fig. S5) appears to be primarily influenced by oceanographic conditions, suggesting a nuanced relationship where environmental factors play a role without serving as definitive barriers to the vertical distribution of SSLs.

## Discussion

### Pelagic seascape insight from sound scattering layer variations

The concept of pelagic seascapes offers a comprehensive framework for understanding the complex issues of scale, patchiness, and change in the pelagic environment^8^. Seascapes are characterized by dynamic and interconnected patterns and processes that operate across a range of spatial and temporal scales^39^. In this framework, SSLs appear as integral components, reflecting the distribution and behavior of micronekton and zooplankton in response to environmental variability at both mesoscale^5,40^ and macroscale levels, as demonstrated in the present study.

The acoustic characterization of the surveyed area provided valuable insights into the distribution and characteristics of SSLs at a macroscale, revealing the diverse patterns of pelagic seascapes across regions. The AT and PA regions exhibited the thickest SSLs, indicating higher micronektonic acoustic backscatter in these regions *vs.* SA. These findings align with prior studies conducted in the Atlantic, as in the Cabo Verde archipelago (between latitudes 11–17°N), which indicated a relatively high micronektonic acoustic backscatter compared to the surrounding waters, illustrating increased density in this region^41^. Proud et al.^17^ reported that high micronektonic acoustic backscatter values were assimilated to high mesopelagic density in the North Atlantic. In our study, the PA also displayed shallow and deep SSLs correlated with areas of the greatest micronektonic acoustic backscatter. The shallow SSL persists near the surface regardless of the diel period. These shallow, persistent SSLs in the PA region studied could be attributed to their high biological productivity^42^, which supports a substantial amount of micronektonic density in the ocean’s upper layers. Furthermore, shallow SSLs in the PA suggest a link to the shallow OMZ. Within OMZs, reduced oxygen levels (hypoxia ~ 60 µmol kg^−1^) influence the distribution and behavior of some marine organisms, including those within SSLs^43^. Several lines of evidence^23,44,45^ suggest that mid-water dissolved oxygen levels restrict the vertical distributions of SSLs, indicating a connection between SSL depths and hypoxia. While our findings revealed a correlation between SSLs and dissolved oxygen levels, the OMZ did not appear to be a limiting factor for the vertical distribution of SSLs. Despite the low oxygen levels in the PA, the SSLs still occupied a wide range of depths.

The SA corresponds to a portion of the North Atlantic Subtropical Gyre. This region is recognized for its distinctive biogeochemical and physical properties, particularly its oligotrophic nature^46,47^. Due to low levels of phytoplankton and primary production in surface waters of oligotrophic regions, these regions are generally considered ocean deserts^47^. SSLs in the SA reflected ecological conditions characterized by nutrient-poor conditions through their relatively shallow, thin, and sparse SSL characteristics. Despite its low marine productivity, the SA exhibits deep SSLs, coinciding with high oxygen levels down to 600 m (~ 150 µmol kg^−1^). This region experiences high dissolved oxygen levels due to the mixing of surface layers and the convergence of currents in the gyre, which affect the position and dynamics of the thermocline. This allows oxygenated surface waters to be effectively mixed downward over several hundred meters. In agreement with previous work highlighting the role of mesoscale dynamics in structuring pelagic ecosystems^32,48^, our findings show that, although anticyclonic gyres are typically oligotrophic oceanic deserts, anticyclonic eddies embedded within them can sustain enhanced mesopelagic backscatter. Previous studies^14,45^ have demonstrated that mesoscale eddies and frontal systems can significantly alter the vertical structure and intensity of mesopelagic scattering layers, making them shallower or deeper, and thereby influencing the distribution and aggregation of micronekton. In the SA, the North Atlantic Subtropical mode water likely contributes to the deep oxygenation by facilitating the downward mixing of oxygenated surface waters.

### Role of Diel vertical migration (DVM) in defining open-sea pelagic seascape

The vertical structure of SSLs across the surveyed regions shows two primary SSLs in the pelagic seascape: the epipelagic and the mesopelagic SSLs. The epipelagic and mesopelagic SSLs exhibited DVM, highlighting the dynamic nature of the pelagic seascape. Specifically, some mesopelagic organisms migrate towards the surface at night, expanding the epipelagic SSL. Conversely, during the daytime, these organisms move downward, expanding the width of the mesopelagic SSLs. This process concurrently leads to a decrease in the thickness of the epipelagic SSLs or the absence of epipelagic layers, as is the case in the Atlantic (SA and AT).

In the southwest Indian Ocean, Proud et al.^17^ identified a surface SSL (0–200 m) in the epipelagic zone, a principal deep SSL (400–600 m), and a secondary deep SSL (750–800 m), both in the mesopelagic zone. On a global scale, Klevjer et al.^22^ identified different types of SSLs based on their vertical distribution and DVM patterns. As in our result, epipelagic SSL and mesopelagic SSL were observed. A noteworthy characteristic of the PA is that not all organisms in the epipelagic layer undertake daytime DVM to greater depths. Some organisms remain at the surface, while others migrate towards the mesopelagic zone. SSLs exhibit a variety of diel vertical patterns: some SSLs migrate upwards or downwards together, others remain stationary at a certain depth, some merge into a single SSL, and others split into several SSLs^17,49^. Elevated values of micronekton acoustic backscatter observed in the epipelagic zone during the night align with known nocturnal vertical migration behavior^50^. Such behaviors highlight the adaptability of micronektonic organisms to different ecological niches within the water column. A noticeable exception occurs in the PA region, where diel differences in the epipelagic SSL are less apparent with overlapping day and night profiles. The PA region is characterized by a persistent surface SSL that remains present day and night, allowing migrants from deeper waters to aggregate within this layer. This persistence likely limits the apparent increase in epipelagic acoustic backscatter despite a pronounced nocturnal decrease in the deep SSL. This suggests a DVM behavior of SSL organisms specific to this region, constrained by hypoxia below a depth of 200 m, where dissolved oxygen levels drop below 60 μmol kg^−1^. Lastly, an intermediate SSL was observed in the PA during the day in the mesopelagic domain (Fig. 2c), showing distinct functional group characteristics of this area and not observed in other surveyed regions.

A major challenge in mesopelagic acoustics lies in distinguishing accurate biological movements from acoustic resonance artefacts, particularly at frequencies below 70 kHz. These artefacts, often due to the diffusion properties of fish swim bladders and backscatter variations with depth, are well-known sources of uncertainty in low-frequency acoustic surveys^33,49,51^. However, our dual-frequency validation confirms the biological nature of the observed DVM, as both the 18 and 38 kHz datasets independently revealed significant vertical migration, with a strong correlation (Supplementary Fig. S6). This validation strengthens the reliability of our single-frequency (38 kHz) DVM results and confirms that the observed acoustic backscatter changes are mainly due to vertical displacement of mesopelagic organisms rather than resonance effects.

While DVM significantly influences SSL dynamics and structuring across regions, it does not diminish the ability of pelagic seascapes to be distinguished based on SSL characteristics. These observations demonstrate that SSL patterns remain reliable indicators of pelagic seascape variability and their ecological organization, despite the complexity introduced by DVM processes.

### Oceanographic drivers shaping the distribution of pelagic sound scattering layer

Oceanographic measurements have been made in regions with contrasting water masses and ocean circulation regimes. The primary water masses encountered in the tropical and subtropical Atlantic regions (AT and SA) are the North Atlantic Central Water (NACW) and South Atlantic Central Water (SACW)^31,52^. The Cabo Verde Frontal Zone separates the NACW and SACW, which are well identified on the SST survey map (Supplementary Fig. S7). The NACW stands out as warmer and more saline than the SACW^53^. These two central water masses are consistently present within the permanent pycnocline, occupying depths ranging from 150 to 600 m and maintaining temperatures exceeding approximately 8 °C. Within the eastern tropical North Atlantic, in the Northwest African upwelling region, SACW is found at depths shallower than 100 m, reaching the euphotic productive zone as depicted by high sea surface chlorophyll-*a* values (Supplementary Fig. S7). Measurements in the PA revealed a shallow thermocline and OMZ, as well as low salinity, and high chlorophyll-*a* concentrations at the surface. These conditions reflect the presence of the Eastern Pacific warm pool, which is the central surface feature of the PA region, with warm, low salinity tropical surface waters above a strong and shallow pycnocline^54^. In the AT, Karstensen et al.^27^ identified the OMZ where oxygen levels dropped to approximately 40 µmol kg^−1^, situated at depths ranging from 400 to 500 m. The OMZ occurred between 300 and 550 m during our study, maintaining relatively higher levels at 80 µmol kg^−1^. Notably, the survey path did not traverse the heart of the OMZ, which might contribute to the observed differences.

The Tropical Pacific Ocean hosts the largest OMZ in the global ocean^55^ with low dissolved oxygen levels between 20 and 50 μmol kg^−1^, a feature never observed in the AT and SA. The vertical extension of OMZ was considerably greater in the PA, with the 60 μmol kg^−1^ contour below the OMZ extending 900–1000 m depth, compared to only 800 m for the higher 120 μmol kg^−1^ contour in the AT. These contrasting oceanographic conditions influence the spatial distribution of acoustic backscatter from micronekton and, consequently, the structure and position of SSLs.

Our study, among others^23,34,56^, confirms that sea temperature, primary production, oxygen, salinity and light availability are predominant drivers in determining the SSL vertical distribution and backscatter intensity and can, therefore, be assumed to shape the pelagic seascape. The vertical distribution of SSLs was predominantly influenced by sea temperature, showing a positive correlation with the shape of the thermocline, except in the PA region, where the thermocline was particularly shallow. Temperature was also the most influential predictor of backscatter intensity at 18 kHz, with higher S_v_ values associated with warmer waters. Chlorophyll-*a* was the second most influential variable for S_v18_, but it emerged as the strongest predictor at 38 kHz in terms of structuring SSL intensity and showing a strong positive relationship with acoustic backscatter. The highest chlorophyll-*a* values have been observed in the PA, coinciding with the highest micronektonic acoustic backscatter. In the epipelagic SSL, peaks of chlorophyll-*a* aligned with or occurred within the SSLs, coinciding with peaks of micronektonic acoustic backscatter. Nevertheless, the low chlorophyll-*a* in the mesopelagic domain did not affect the SSL vertical distribution. The dissolved oxygen level was a significant variable influencing micronektonic acoustic backscatter intensity and vertical distribution. Dissolved oxygen also showed a positive relationship with acoustic backscatter, indicating that higher oxygen levels promote greater micronekton abundance, particularly at 38 kHz. Interestingly, our results do not fully support the hypothesis of oxygen limitation^23,44^, as SSLs were observed not only in shallow oxygenated waters but also in the hypoxic zone at depths of approximately 800 m in the PA region. Salinity positively influenced the vertical position of SSL and micronektonic acoustic backscatter. The significant effects of geographical location, particularly in the Sargasso and Pacific regions, suggest that the micronektonic distribution and acoustic characteristics are not uniform in all marine regions.

These findings align with previous macro-scale studies that identified similar factors as primary influences on SSL distribution^23,33^. Numerous studies have highlighted the role of sea temperature in shaping the distribution of SSLs^44,56,57^. Furthermore, Boersch-Supan et al.^58^ and Diogoul et al.^37^ have demonstrated the relationship between the depths of SSLs and the presence of thermal and density gradients. The thermocline depth is closely associated with the habitat and population levels of zooplankton organisms and serves as an ecological boundary for pelagic organisms^59^. Regarding phytoplankton biomass, our results are consistent with studies showing that enhanced primary production increases micronekton populations^30,33^. The alignment of phytoplankton biomass peaks with SSLs over the continental shelf is also consistent with previous observations^37^. Dissolved oxygen levels affect the cycles of carbon and nutrients, as well as the life cycles of marine organisms^60,61^. There is a relationship between oxygen levels and the depth of SSLs, suggesting that OMZs constrain the vertical extent of SSLs^22,23,44^. This phenomenon is observed in the Peruvian^34^ and the Californian^44^ coastal upwelling systems. Reduced oxygen levels constrain the vertical distributions of plankton and fish^22^ and provide a refuge from predators^62^.

Similarly to our results in the PA, Klevjer et al.^22^ have shown that most micronektonic acoustic backscatter is located deep in the hypoxic zone, suggesting that hypoxia avoidance may not be a general factor in the vertical distribution of SSLs. It has been hypothesized that mesopelagic organisms have developed various mechanisms to lower their metabolic rates, enabling them to endure hypoxic conditions during the daytime^63,64^. Salinity had a significant effect on SSL micronektonic acoustic backscatter at both frequencies and also influenced their vertical distribution, consistent with studies that highlight the importance of habitat salinity in the distribution of marine species. There are notable associations between salinity levels and the composition of zooplankton and micronekton communities^65^. In the Pacific, La et al.^66^ established a link between salinity and micronektonic acoustic backscatter, with rising salinity levels associated with increased micronektonic acoustic backscatter. Zooplankton, a major component of micronektonic biomass, displays diverse responses to haloclines, including positioning itself near the halocline and migrating through it^66–68^. Our findings suggested that the halocline influences the SSL vertical variation even without constraints on its vertical extension. PAR significantly influenced SSL intensity at both frequencies, consistent with a light-induced diel vertical migration behavior.

Light availability regulates the vertical positioning of SSL, as organisms descend to darker depths during the day and ascend at night to feed in the productive surface layers^35,69^. The significant effect of PAR on backscatter intensity is consistent with diel vertical migration patterns confirmed by our analysis, reflecting differences in SSL depth and backscatter intensity between day and night. This is consistent with the principle that mesopelagic organisms occupy “light comfort zones” at specific isolumes balancing predation risk and foraging efficiency^35,70^. Importantly, PAR provides a mechanistic explanation for the strong relationship between chlorophyll-*a* and SSL that we observed: higher phytoplankton biomass increases light attenuation, thereby shoaling the euphotic zone and, consequently, the isolume depths that define the mesopelagic habitat. Rather than indicating direct trophic interactions between phytoplankton and mesopelagic communities, the chlorophyll-*a* effect reflects an optical control of the vertical habitat through light absorption. This light-mediated framework is also supported by observations that mesopelagic organisms respond rapidly to even transient light variations due to cloud shadows^71^, demonstrating the sensitivity of these communities to the light regime and validating light as a driver of SSL structuring, alongside physical stratification and biogeochemical factors.

Furthermore, subsurface mixing in the AT region, characterized by strong surface currents and vertical shear (Supplementary Table S1; Supplementary Fig. S8), exerts intricate effects on micronektonic biomass, significantly impacting their vertical distribution and acoustic backscatter. Diogoul et al.^37^ emphasize the role of stable physical conditions in SSL formation, highlighting the complex relationship between ocean dynamics and biological processes. Among these dynamics, shear plays a significant role in structuring plankton patches in the ocean^72^. Vertical shear, in particular, can drive SSL formation by interacting with plankton behavior, with particular zooplankton species exhibiting behavioral responses to shear^73^. Zooplankton that migrate vertically become entrapped in regions of elevated shear due to the interaction between their physical characteristics and fluid motion^74^.

Dynamic oceanographic features, such as currents and eddies, as well as the interaction of water masses, significantly shape the pelagic seascapes that underpin SSL distributions. Mesoscale eddies are closely linked to physical ocean fields (including sea temperature, salinity, heat flux, heat content, and vertical mixing) and also modulate biogeochemical processes such as nutrient distribution and plankton dynamics^75–77^. Our eddy detection revealed anticyclonic eddies in the SA, consistent with the observed deepening of the SSL in this region (Supplementary Fig. S5), as well as cyclonic eddies in the AT and a large cyclonic eddy in the PA, where a strengthened and shallow SSL was observed. Mesoscale eddies modify the vertical structure of the ocean through rotational circulation, altering thermocline depth, stratification, and nutrient-oxygen distribution^78,79^. In the northern hemisphere, cyclonic vortices rotate counterclockwise and anticyclonic vortices rotate clockwise, as observed in our result. Anticyclonic eddies lower the thermocline through downwelling, which limits nutrient upwelling and creates oligotrophic nuclei^76^. In contrast, cyclonic eddies lower the thermocline through upwelling, thereby enhancing nutrient injection and primary productivity^79^. Our individual eddy scale analysis provides direct evidence that mesoscale eddies restructure pelagic habitats. In the anticyclonic case, high S_v_ values near the eddy center suggest retention due to sinking and internal accumulation of SSLs organisms, consistent with the trapping and “biological oasis” behavior described for warm-core eddies^14^. In contrast, the cyclonic example revealed a reduction in S_v_ in the eddy center but stronger backscattering along the periphery, reflecting upwelling, outward transport, and increased biological activity at the periphery of the eddy. These mesoscale dynamics associated with geostrophic circulation generate spatially heterogeneous habitats that modulate the abundance, behavior, and vertical positioning of marine species^14,33,45,48^. This variability contributes to the complexity of pelagic seascapes and underscores the importance of understanding these dynamics using SSLs. The interplay between physical drivers, such as temperature gradients, oxygen minima, and salinity variations, and biological factors, including phytoplankton production, underscores the complexity of SSL formation and persistence. Understanding these dynamics is critical for predicting the response of SSLs to environmental changes, particularly in light of climate-driven oceanographic shifts and increasing anthropogenic stressors. Enhanced knowledge of these processes, using SSL to define pelagic seascapes, will improve our ability to assess marine ecosystem changes and support better resource management within dynamic ocean environments.

## Methods

### Study area

**Fig. 6:**
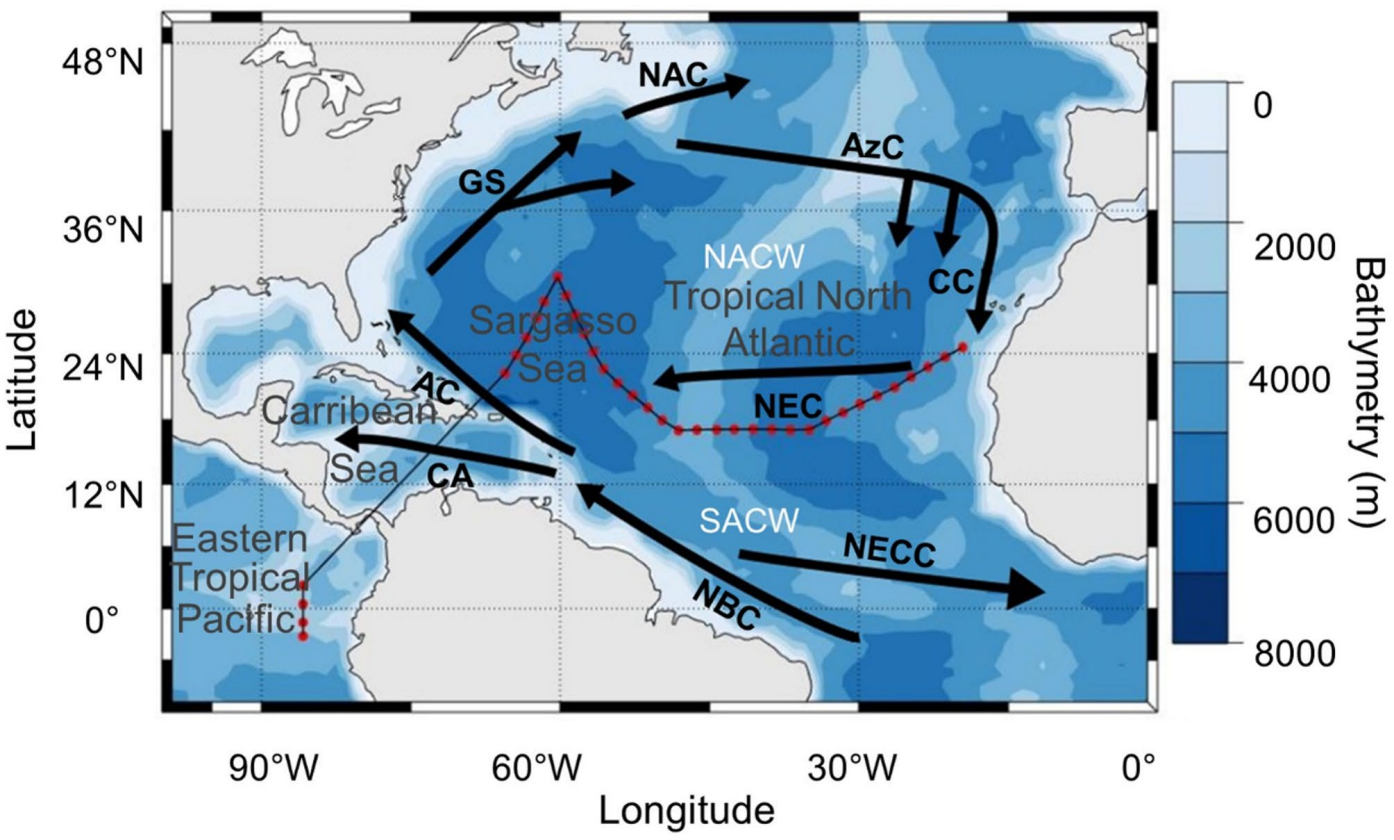
Survey design (black line) with CTD stations (red points) of SO287-CONNECT sea cruise from North West Africa (Canary Islands) to the Eastern Pacific Ocean crossing the Panama Canal. Black arrows are schematic representations of oceanic currents surface: Canary Current (CC), North Atlantic Equatorial Current (NEC), North Atlantic Equatorial Counter Current (NECC), North Brazil Current (NBC), Caribbean Current (CaC), Antilles Current (AC), Gulf Stream (GS), North Atlantic Current (NAC), and Azores Current (AzC). Water masses: North Atlantic Central Water (NACW) and South Atlantic Central Water (SACW) (adapted from Tomczak and Godfrey^82^).

The SO287 Connect cruise was carried out on-board the research vessel R/V SONNE along a transect (Fig. 6) from the Canary Islands (Las Palmas, Spain) to Ecuador (Guayaquil), crossing the Eastern Tropical North Atlantic Ocean (AT), the Sargasso Sea (hereafter SA), the Caribbean Sea and the Eastern Tropical Pacific Ocean (hereafter PA). The research cruise, conducted from December 11, 2021, to January 11, 2022, traversed various oceanographic zones with distinct characteristics. Major currents, such as the North Atlantic and Canary Currents, influence temperature and salinity patterns in the Northeast Atlantic, with upwelling phenomena off West Africa supporting marine productivity^40,80^. The surveyed region encompasses the North Atlantic (NA) gyre, delimited by the Canary Current to the east and the North Equatorial Current (NEC) to the south, connecting the west coast of Northern Africa with the east coast of Central America. The Canary Current feeds the Eastern Boundary Upwelling system (EBUS) on the African coast, where nutrient-rich deep water fosters high primary production, fueling the food web. The SA in the center of the NA gyre features oligotrophic conditions with lower nutrient concentrations^46^. Significant oceanic and atmospheric interactions, including equatorial upwelling and the influence of El Niño and La Niña events, mark the Eastern Tropical Pacific. These phenomena contribute to the region’s complex ocean dynamics, affecting weather patterns and marine ecosystems far beyond the Pacific^81^.

The NEC flows westward in the latitudinal range of about 8–18°N in the Tropical Atlantic Ocean^83^. This current is vital for translocating heat and salt across the Atlantic, influencing the thermohaline circulation. Flowing eastward between 5°N and 10°N latitude, the North Equatorial Counter Current (NECC) acts as a dynamic boundary between the NEC and the equatorial currents, playing a critical role in the distribution of equatorial water masses and heat.

The Caribbean Current (CaC) carries water westward through the Caribbean Sea, contributing to the regional circulation and influencing the climate and marine conditions of the surrounding coastal regions. Further north, the powerful and warm Gulf Stream (GS) transports water north-eastward along the U.S. East Coast. This current is one of the strongest ocean currents in the world and is crucial for moderating the climate in Western Europe. As the Gulf Stream continues its journey, it becomes the North Atlantic Current (NAC), which extends across the Atlantic, carrying warm waters towards Europe and influencing the weather patterns of the North Atlantic region^84^. Near the Azores Islands, the Azores Current (AzC) branches off eastward from the Gulf Stream. This current is a significant component of the subtropical gyre circulation and influences the oceanographic conditions of the Azores region^85^. Additionally, the North Brazil Current (NBC) flows northwestward off the Brazilian coast, which is crucial in transferring warm water and heat from the South Atlantic to the North Atlantic^86^.

### Data collection

#### Acoustic data

Acoustic data were recorded from November 12, 2021, to January 11, 2022. Hydroacoustics data were continuously recorded (24/24) along the cruise track using a Simrad EK60 hull-mounted echosounder operating at 18, 38, 120 and 120 kHz. The EK60 was set at 20 log R time-varied gain function (where R is the range in meters), using a pulse length of 1.024 ms and maximal transmission power of 2000 W. Acoustic data were processed with an offset of 10 m below the sea surface to avoid integrating air bubbles during post-processing analysis and considering echosounder depth immersion.

Acoustic backscatter and water velocities were recorded continuously along the path cruise using a hull-mounted Acoustic Doppler Current Profiler (ADCP), Teledyne RD Instruments 75 kHz Ocean Surveyor ADCP. The ADCP operated in narrowband mode, utilizing 8-m bins with an 8-m blanking distance. Each pulse lasted 1.45 s, resulting in 100 bins recorded. ADCP data was archived on board using the Teledyne RD Instrument’s software Vessel-Mounted Data Acquisition System (VMDAS), which continuously merges the ADCP data stream with external data streams containing ship navigation and attitude information. VMDAS internally converts ADCP data from beam coordinates to Earth coordinates. All data are provided as single-ping ensembles in binary format^87^.

#### Environmental data

Hydrographic data were collected using a rosette sampler coupled with a Seabird 911plus probe, featuring calibrated sensors for water conductivity (SBE 4C), temperature (SBE 3plus), and depth (CTD). The CTD was also equipped with a fluorescence sensor to measure chlorophyll-*a* concentration (WetLabs ECO), a proxy of phytoplankton biomass, a sensor for dissolved oxygen (µmol kg^−1^; SBE 43), and a photosynthetically active radiation (PAR) sensor (µmol m^−2^ s^−1^) to measure in situ light availability throughout the water column. CTD casts were performed along the cruise track at 36 stations (Fig. 6; no data are available in the Caribbean Sea). The fluorescence sensor calibration was performed using high-performance liquid chromatography (HPLC). The oxygen sensors of the CTD were calibrated using Winkler titrations. The CTD cast deployment consisted of a shallow station down to 1,000 m during the day, between 12:00 and 14:30 local time, at the solar zenith, and a night station between 23:30 and 03:00. Diurnal stations in the Pacific were excluded from analyses due to potential non-photochemical quenching (NPQ) effects identified through HPLC calibrations. Chlorophyll maxima in the Pacific were shallower and more sensitive to NPQ under high solar radiation, resulting in biased fluorescence measurements at diurnal stations compared to Atlantic stations, which showed strong correlations between CTD fluorescence and HPLC-derived chlorophyll-a.

Additionally, environmental data were collected from satellite remote sensing products, including daily Sea Surface Chlorophyll (SSC), Sea Surface Temperature (SST), and Sea Level Anomaly. Survey maps of SSC and SST data were created from the daily daytime series of the AQUA-MODIS sensor with a 4 km resolution (available at https://oceancolor.gsfc.nasa.gov/).

Daily level 4 Gridded Absolute Dynamic Topography and associated geostrophic velocity components at a spatial resolution of 1/4° were obtained from the Copernicus Marine Environment Monitoring Service (CMEMS, 10.48670/moi-00148). Mesoscale eddies were identified and tracked using the Angular Momentum Eruption Detection and Tracking Algorithm (AMEDA)^88^, which detects eddy centers through local normalized angular momentum extremes and delineates boundaries using mean velocities along 0.2 cm sea surface height contours. Eddy tracking used a nearest-neighbor approach with a maximum propagation velocity of 6.5 km d^−1^ and a 10-day search window for temporarily undetected eddies.

### Data processing

The analyses were performed exclusively on the 18 and 38 kHz frequencies. These frequencies offer the necessary depth penetration and reduced acoustic attenuation to cover the mesopelagic layer^16^ effectively. Higher frequencies (120 and 200 kHz) were excluded due to their limited effective range in deeper waters^89^. The 18 and 38 kHz frequencies are among the most commonly used in mesopelagic research, where they are employed to characterize SSLs, quantify micronekton distributions, and examine their vertical migration dynamics^17,90^.

**Fig. 7:**
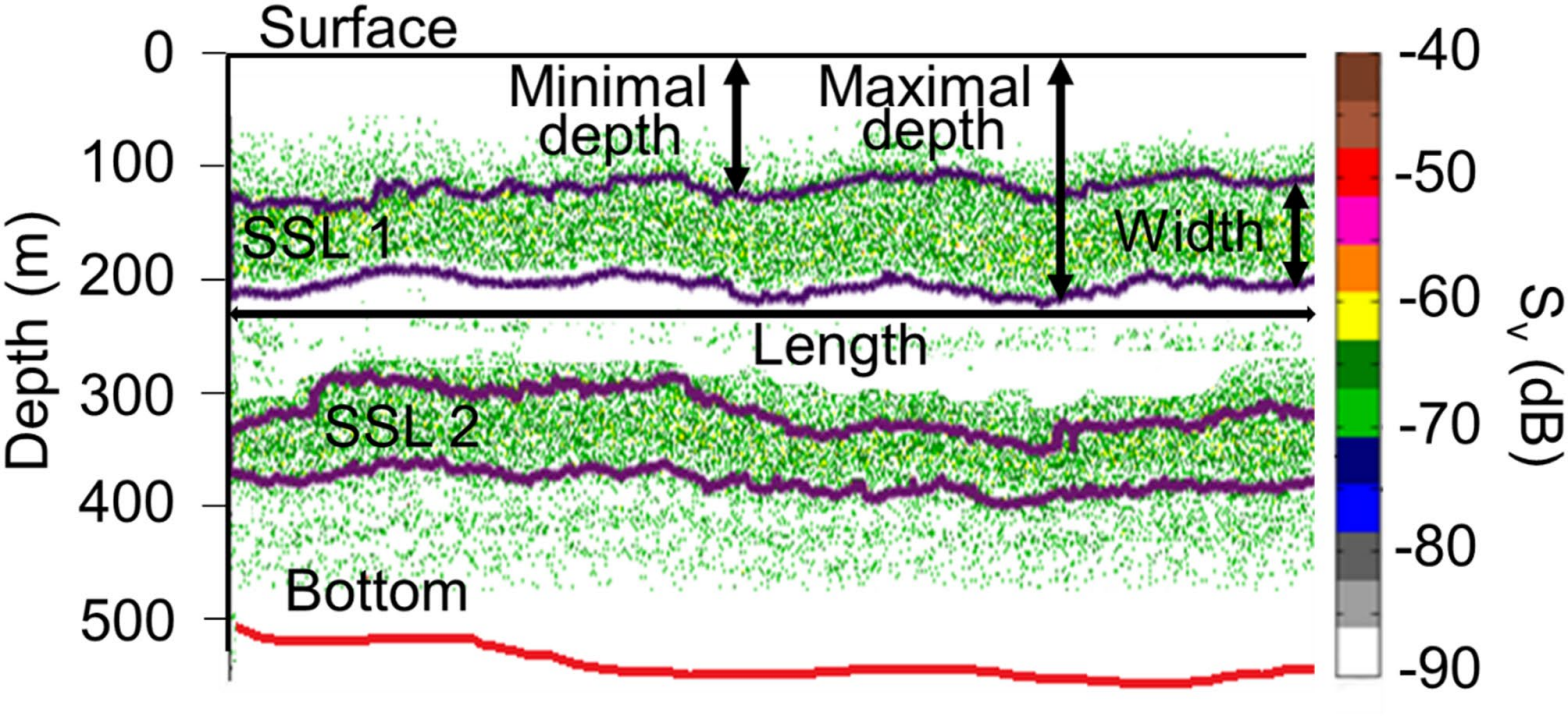
Schema showing two sound scattering layers (SSLs). SSL descriptors extracted by Matecho^91^ from acoustic echograms were maximum depth, minimum depth, vertical width, and length (in meters). The right axis depicts mean volume backscattering strength (S_v_ in dB), and the left axis depicts local bottom depth (in meters). The sea surface is represented by a black line (0 m) and the bottom by a red line.

EK60 acoustic data was processed using the in-house tool Matecho^91^. Matecho is an integrative processing software that enables the correction of echograms, specifically bottom depths, by removing empty pings and echogram interference, as well as reducing background noise. It offers several other functionalities, including automatic data correction and filtering, echo-integration and extraction of fish schools and SSL. We utilized temperature and salinity data (from the CTD data) to perform sound speed corrections on our acoustic measurements, thereby addressing variations in sound speed at different depths^92^. After correction, acoustic data were echo-integrated per 0.1 nautical miles (nmi). The mean acoustic volume backscattering strength (S_v_ in dB; hereafter referred to as micronektonic acoustic backscatter) was used to quantify relative acoustic density as a proxy for marine organism abundance, recognizing that this measure reflects the intensity of acoustic backscatter rather than direct estimates of micronekton biomass. After data echointegration, SSLs with mean S_v_ values above the threshold of − 70 dB were extracted, and SSL descriptors were calculated (Fig. 7): minimum depth (m), maximal depth (m), width (m), and mean S_v_ (dB)^93^.

The SSL extraction implemented in Matecho is based on segmenting the echo integration from a given threshold on echo levels (calculating isocontour according to the selected S_v_ threshold and Matlab algorithm “contourf.m”). In addition to SSL descriptors for both 18 and 38 kHz, the Weighted Mean Depth (WMD) was calculated^22^. The WMD is a proxy for the depth of the greatest acoustic density to assess the vertical distribution of organisms. To confirm that the patterns of vertical WMD migration reflected accurate biological behavior rather than artefacts of acoustic resonance, we conducted cross-frequency validation. This involved estimating the WMDs independently at 18 kHz and 38 kHz, assessing their Pearson correlation and comparing the migration amplitudes between day and night (Supplementary Fig. S6).

### Data analysis

Acoustic descriptors were used for different but complementary purposes within the analysis: multiple SSL metrics were extracted to support seascape classification and vertical structure analyses, whereas environmental driver modelling focused exclusively on acoustic backscatter intensity (S_v_).

**Fig. 8:**
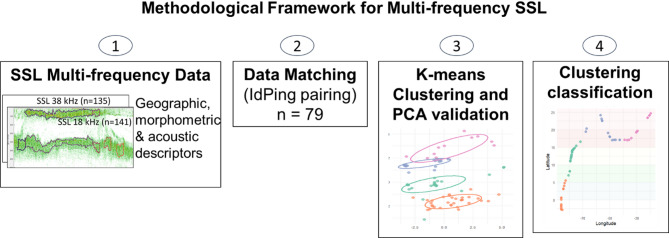
Methodological framework for the classification of pelagic seascapes.

To characterize SSL pelagic seascape (Fig. 8), a K-means clustering analysis was performed on an integrated set of geographic, morphological, and dual-frequency (18 and 38 kHz) acoustic descriptors using the ‘stats’ package in R Studio to group similar descriptors. For direct comparability between frequencies, the extracted SSLs were matched at the ping level to ensure that the 18 kHz and 38 kHz measurements sampled the water volume simultaneously and at the same location. Geographical and morphometric descriptors defined the spatial and bathymetric context of SSL habitats, while bi-frequency acoustic metrics quantified their backscatter, intensity, and frequency-dependent signatures, which are indicative of gas-bearing organisms. The optimal number of clusters (n) for the K-means analysis was determined using silhouette widths. This approach measures the difference between intra-cluster similarity and similarity with the nearest cluster^94^.

The mean S_v_ was used to analyse DVM. Diel changes of S_v_ (Δ_DVM_) were first studied by comparing mean S_v_ for day *vs* night in the vertical dimension (i.e., the mean value for each 1 m depth cell). Diel transition periods were removed from analyses to avoid biases caused by DVM^37,93^. These transition periods are identified based on the sun’s altitude, specifically around sunset and sunrise, when the sun’s altitude is between ± 18°^24^. Accordingly, daytime and night-time periods were defined as solar altitudes above + 18° and below − 18°, respectively. This ensures that any changes in SSL metrics are not attributed to diel transition periods.

Environmental driver modelling focused exclusively on S_v_ at 18 and 38 kHz, while other SSL descriptors were used for seascape classification and for analysing vertical structure and diel vertical migration.The relationship between acoustic metrics and environmental variables was investigated using Generalized Additive Models (GAMs^95^). GAMs are well adapted for modelling non-linear relationships between acoustic and environmental variables^56,96^. GAMs allow the inclusion of correlation structures to model the inherent autocorrelation of acoustic survey data. Models were selected using Akaike’s information criterion^97^. The S_v_ models at 18 and 38 kHz were fitted using an identity link function and Gaussian errors. S_v_ observations used in the GAM analyses include both daytime and nighttime measurements, which were pooled to capture the full range of environmental conditions experienced by SSLs. In addition to environmental variables such as sea temperature, salinity, dissolved oxygen, and chlorophyll-*a* we have included geographic position (location) and PAR in the models due to their potential to influence SSL distribution^37^. Each environmental variable was matched to the corresponding acoustic data based on the time and location of the measurements. Specifically, we identified the corresponding SSL data for each CTD station by locating the data within a 0.1 nautical mile (0.1 nmi) radius around each CTD station. This matching procedure ensured that the dataset included S_v_ measurements and their associated environmental conditions from both daytime and nighttime periods. Water turbidity showed a robust positive correlation with chlorophyll-*a* (r = 0.8, Supplementary Fig. S9). Moreover, turbidity primarily reflects the physical properties of water, such as suspended particles, water color, and suspended sediments. In contrast, chlorophyll-*a* provides insights into the biological and chemical composition of the water column. Consequently, turbidity was excluded from the model. However, despite a strong correlation between sea temperature and dissolved oxygen (r = 0.8, Supplementary Fig. S9), the model retained both variables due to their significant relevance in the pelagic ecosystem. Water temperature and dissolved oxygen are crucial factors that influence marine ecosystems. The validity assumptions of the models were then assessed by checking for normality of distributed errors and homogeneity of residuals (Supplementary Fig. S10). To accommodate the disparity in units among variables, a transformation or standardization process was applied before constructing the models. This was undertaken to ensure that all variables were normalized to a uniform scale, enabling precise comparisons and interpretations within the model. Thus, the final S_v_ models used were:

1 $${\mathrm{S}}_{{{\mathrm{v18}}}} = {\mathrm{Temp}}. + {\mathrm{s}}\left( {{\mathrm{Chla}}.} \right) + {\mathrm{s}}\left( {{\mathrm{O}}_{{2}} } \right) + {\mathrm{Sal}}. + {\mathrm{s}}\left( {{\mathrm{PAR}}} \right) + {\mathrm{Location}} +\upvarepsilon$$

2 $${\mathrm{S}}_{{{\mathrm{v38}}}} = {\mathrm{Temp}}. + {\mathrm{s}}\left( {{\mathrm{Chla}}.} \right) + {\mathrm{s}}\left( {{\mathrm{O}}_{{2}} } \right) + {\mathrm{Sal}}. + {\mathrm{PAR}} + {\mathrm{Location}} +\upvarepsilon$$

where “s” is the smooth functions, “Location” is the discriminated zones (i.e., AT, SA and PA), and ε is the error term in the model. Temp.: sea water temperature (°C); Chla.: Chlorophyll-*a* (µg m^−3^); Sal.: Salinity (PSU); O_2_: dissolved oxygen (μmol kg^−1^); PAR.: Photosynthetic Active Radiation.

Along the SO287 Connect survey, no CTD stations were performed in the Caribbean Sea; thus, we have excluded this data from the modelling exercise (Supplementary Fig. S11).

## Supplementary Information

Below is the link to the electronic supplementary material.

Supplementary Material 1

## Data Availability

The acoustic and ADCP datasets analyzed during the current study are available in PANGEA repositories. Acoustic data: 10.1594/PANGAEA.975986; ADCP data: 10.1594/PANGAEA.956601.
