# Supplementary material for: Characterisation of pelagic seascapes through micronektonic and zooplanktonic scattering layers
Source: Sci Rep. 2026 Jan 23;16:6378. doi: 10.1038/s41598-026-36104-1 (PMC12910103; doi:10.1038/s41598-026-36104-1)
Supplement: Supplementary file 1 — Supplementary Material 1 [file 41598_2026_36104_MOESM1_ESM.docx]

# supplementary material


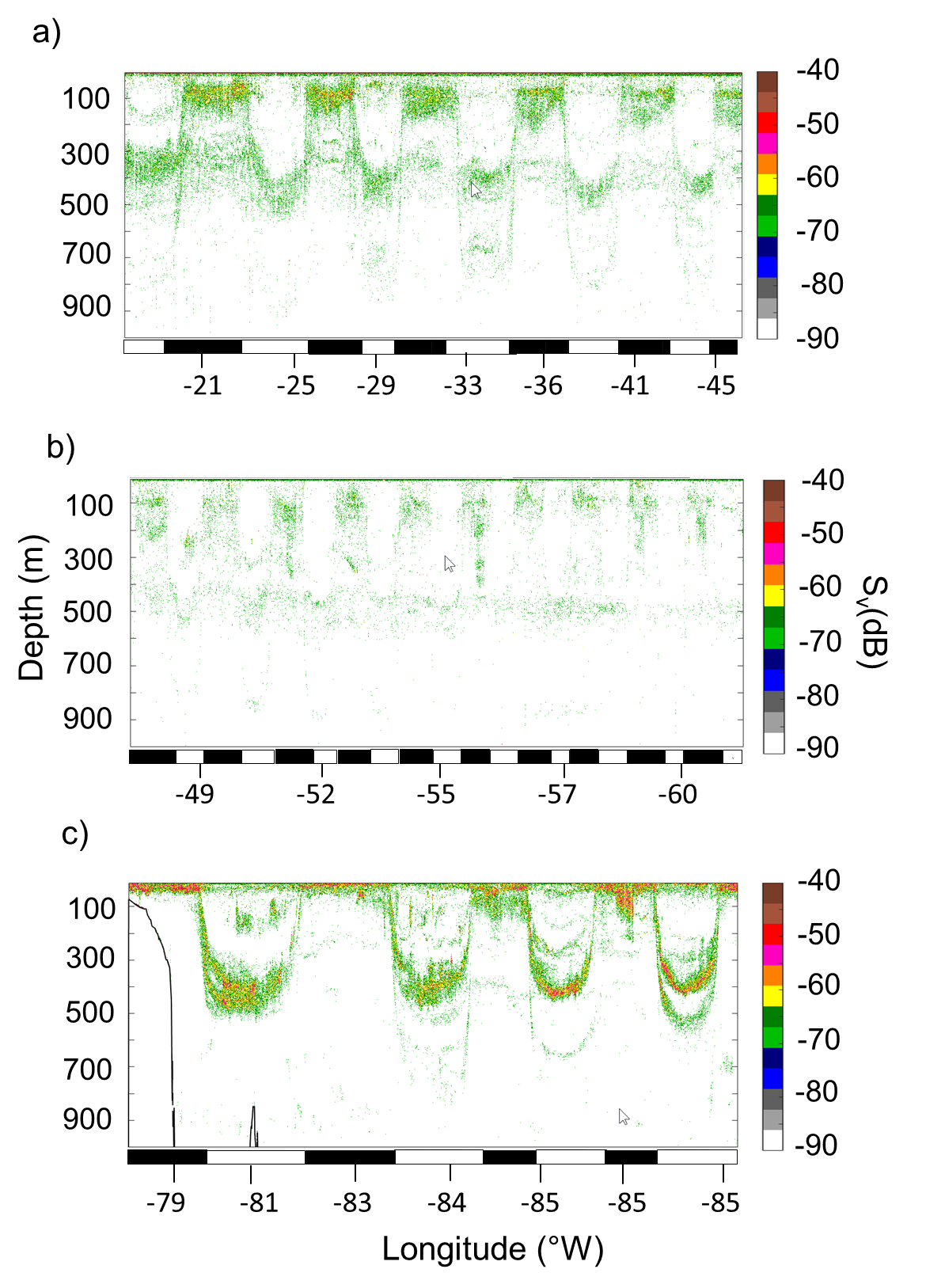


**Supplementary Fig. S1:** Echograms of micronektonic acoustic backscatter (S_v_ in dB) at 18 kHz illustrating their diel vertical migration in three tropical regions. (a) Eastern Tropical North Atlantic Ocean (AT), (b) Sargasso Sea (SA), and (c) Eastern Tropical Pacific Ocean (PA). The white and black rectangle below the echogram shows day and night periods.


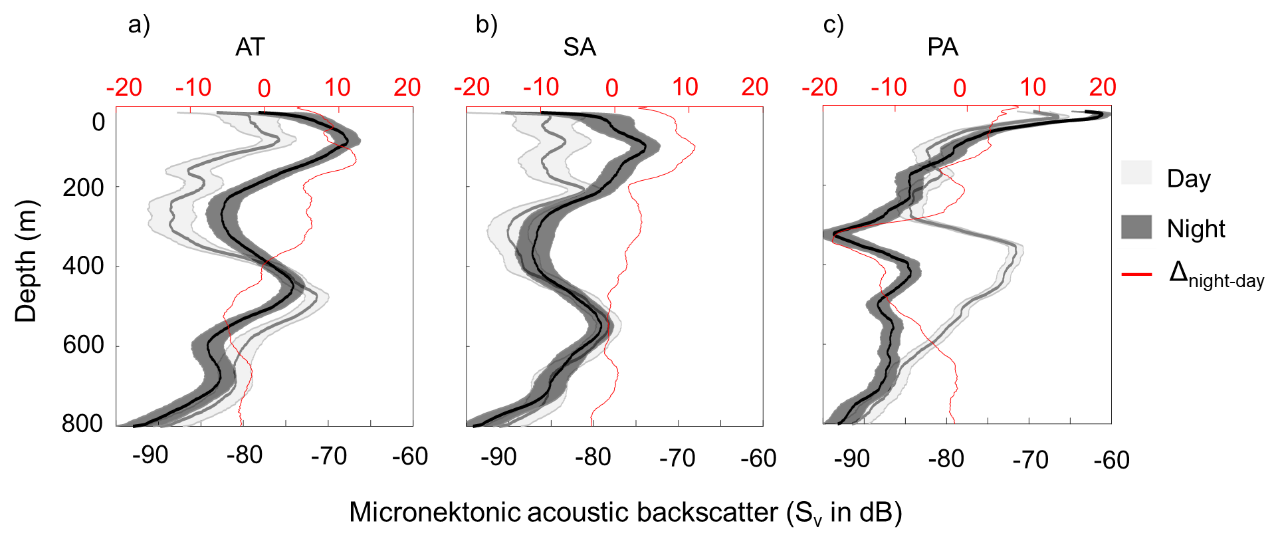


**Supplementary Fig. S2:** Daytime (grey) and nighttime (dark) mean vertical profile of micronektonic acoustic backscatter (S_v_ in dB) at 18 kHz within the water column in three intertropical regions. The difference between day and night ∆night-day (red line and x-axis) illustrates the diel vertical migration. (a) the Eastern Tropical North Atlantic Ocean (AT), (b) Sargasso Sea (SA) and (d) Eastern Tropical Pacific Ocean (PA). The shaded areas represent the standard deviation.


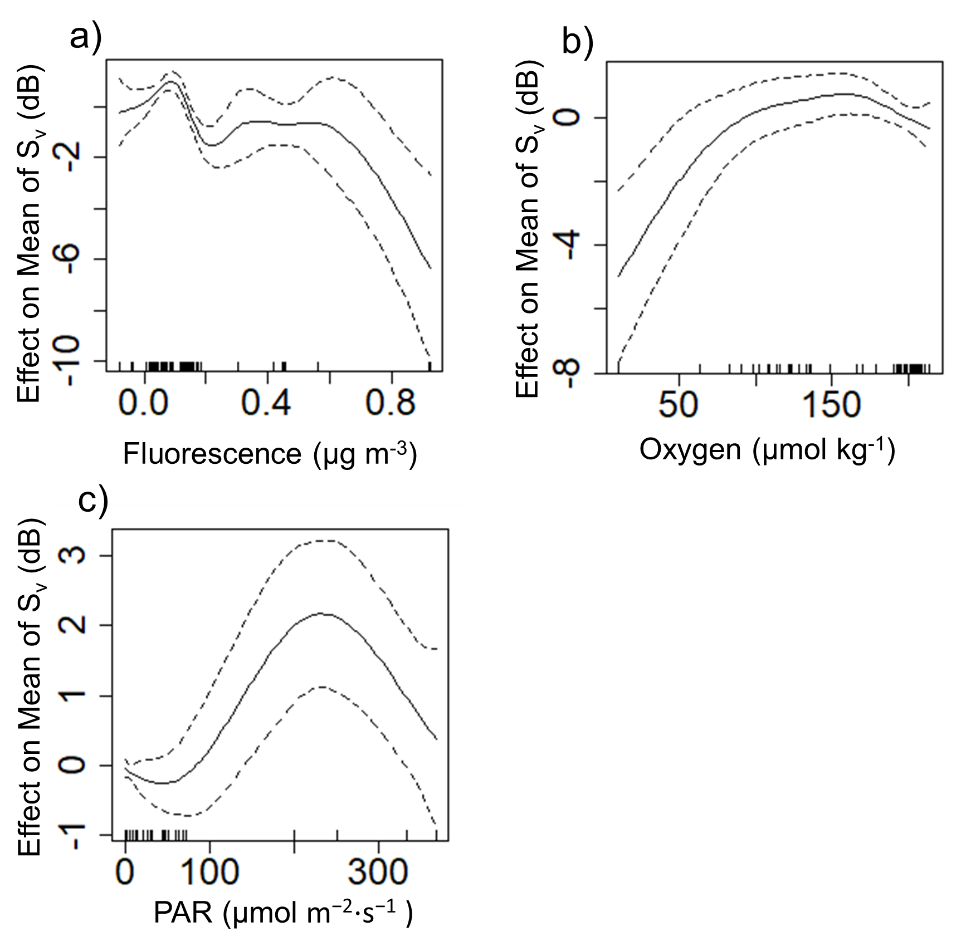


**Supplementary Fig. S3:** Generalized additive model terms showing the effect of environmental continuous variables [(a) fluorescence, (b) dissolved oxygen, (c) Photosynthetically Active Radiation on the mean volume backscattering strength (S_v_, in dB) of the Sound Scattering Layer (SSLs) detected at 18 kHz. The solid lines are the estimates of the smooths, and the dotted lines are the standard errors of the estimated smooths, considering the error in the model intercept.


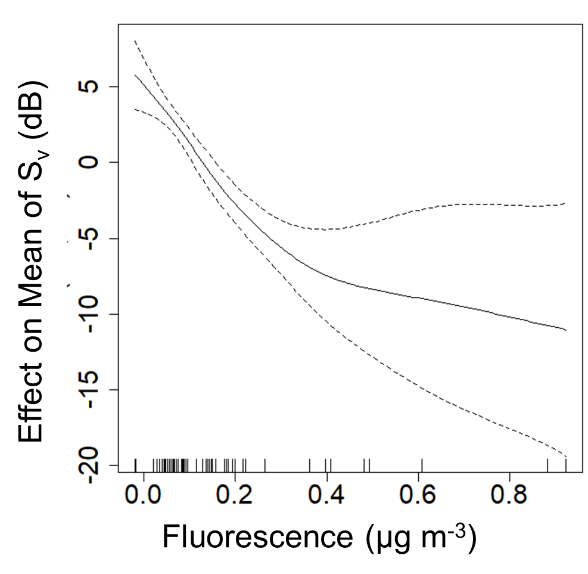


**Supplementary Fig. S4**: Smooths of generalized additive model terms showing the effect of fluorescence on the mean volume backscattering strength (S_v_, in dB) of Sound Scattering Layer (SSLs) detected at 38 kHz. The solid lines are the estimates of the smooths, and the dotted lines are the standard errors of the estimated smooths, considering the error in the model intercept.

**
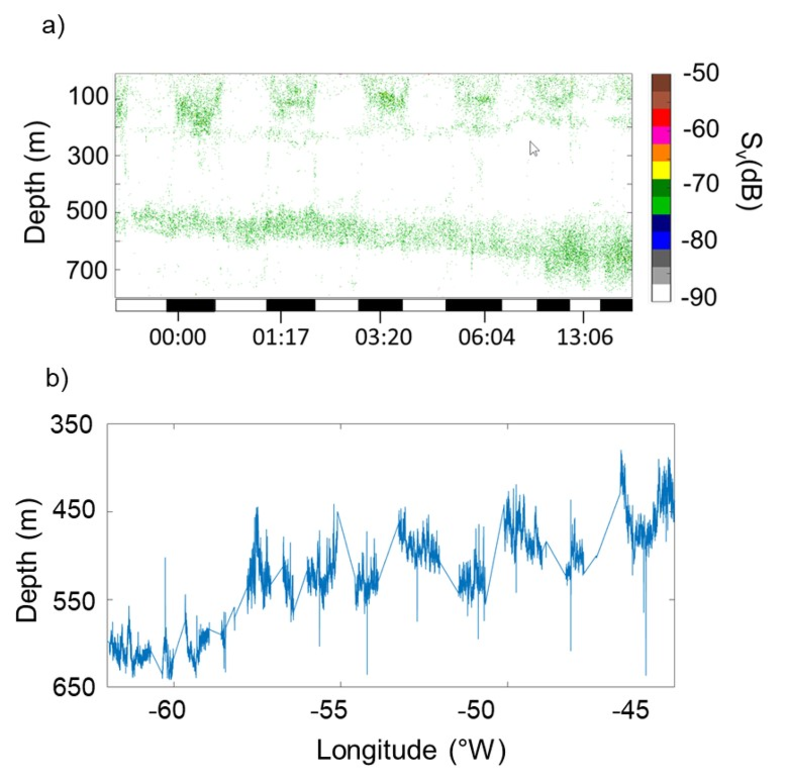
**

**Supplementary Fig. S5**: (a) Echogram (38 kHz) observed in the Sargasso Sea (SA) depicting a regular deepening and widening of the mesopelagic micronektonic layer from 45°W to 62°W. The bottom panel represent the diel period: day (white), night (black). (b) Plot mean of micronektonic acoustic backscatter, *i.e*., volume backscattering strength (S_v_, in dB) of sound scattering layers (SSL) echo-integrated per 0.1 nautical miles (nmi), showing the deepening of the mesopelagic SSL from 400 to 650 m in the Sargasso Sea.


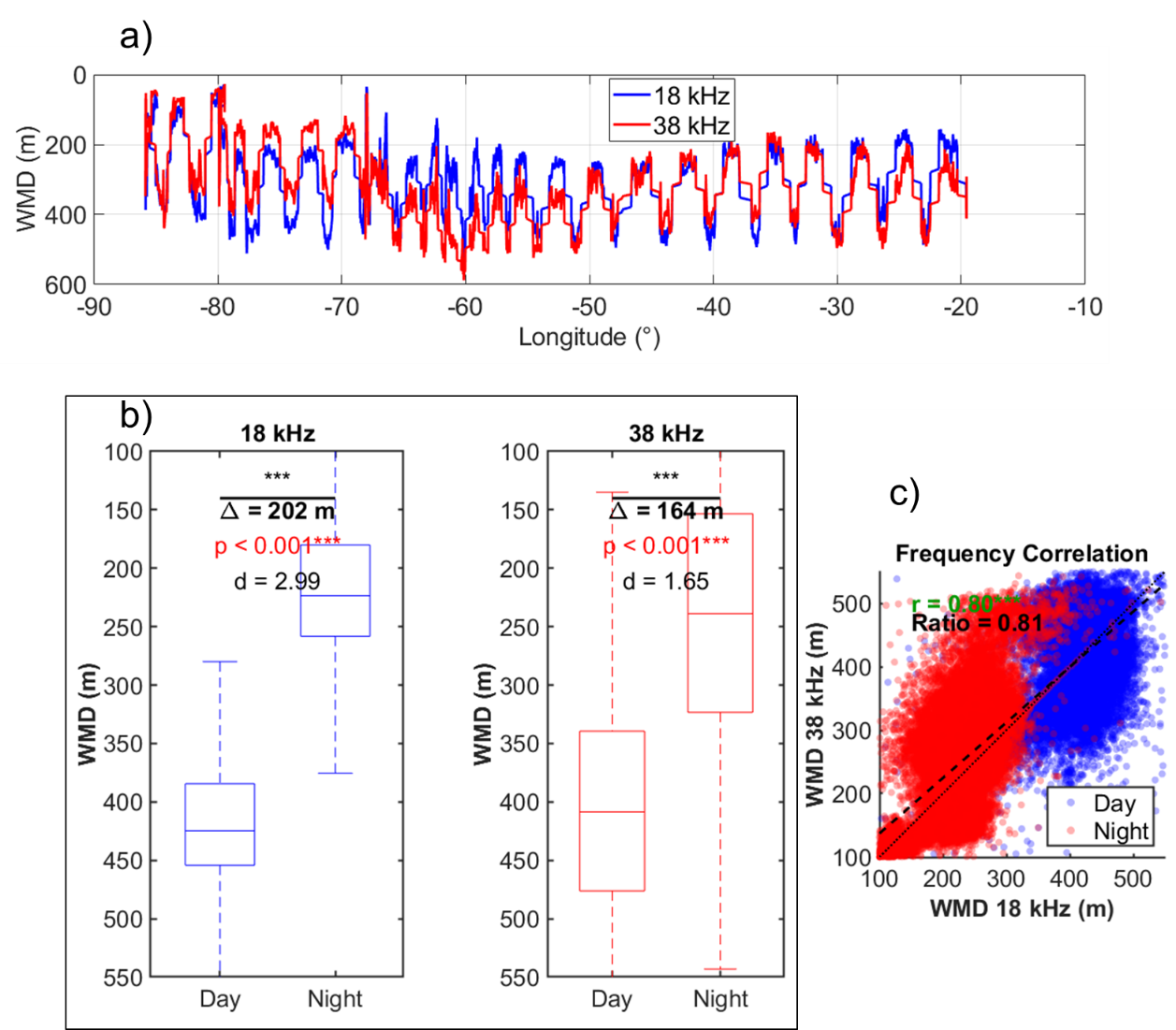


**Supplementary Fig. S6**: Cross-frequency validation of weighted mean depth (WMD) patterns: a) Spatial WMD profiles at 18 kHz (blue) and 38 kHz (red) along the transect; b) Diel vertical migration at both frequencies; c) Correlation between WMD₁₈ and WMD₃₈ (migration amplitude ratio = 0.81). Daytime (red) and nighttime (blue) samples; the dotted line indicates the relationship.


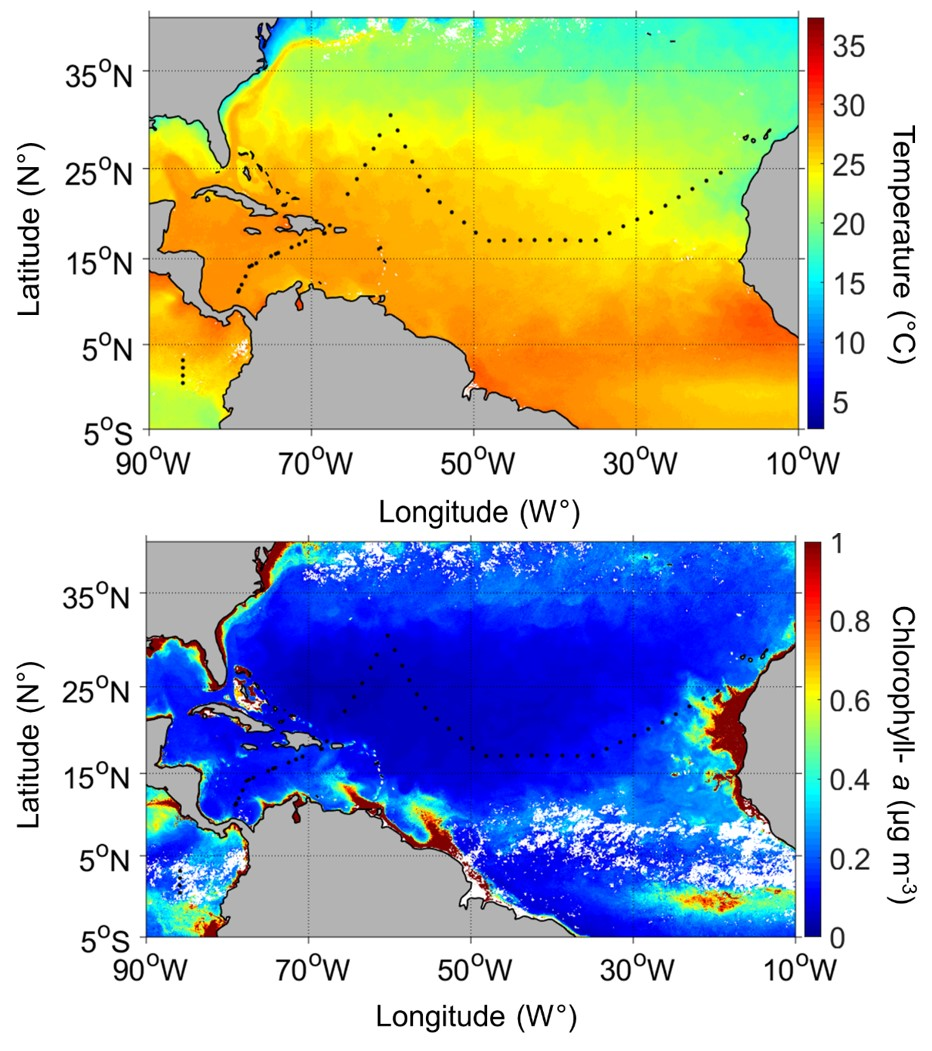


**Supplementary Fig. S7.** Map of Sea Surface Temperature (SST) (top) and Sea Surface Chlorophyll-*a* (SSC) (bottom) daily averages across the study area during the Connect cruise from December 10, 2021, to January 10, 2022. Black points depict the cruise track; Source NASA (<https://oceancolor.gsfc.nasa.gov/>).


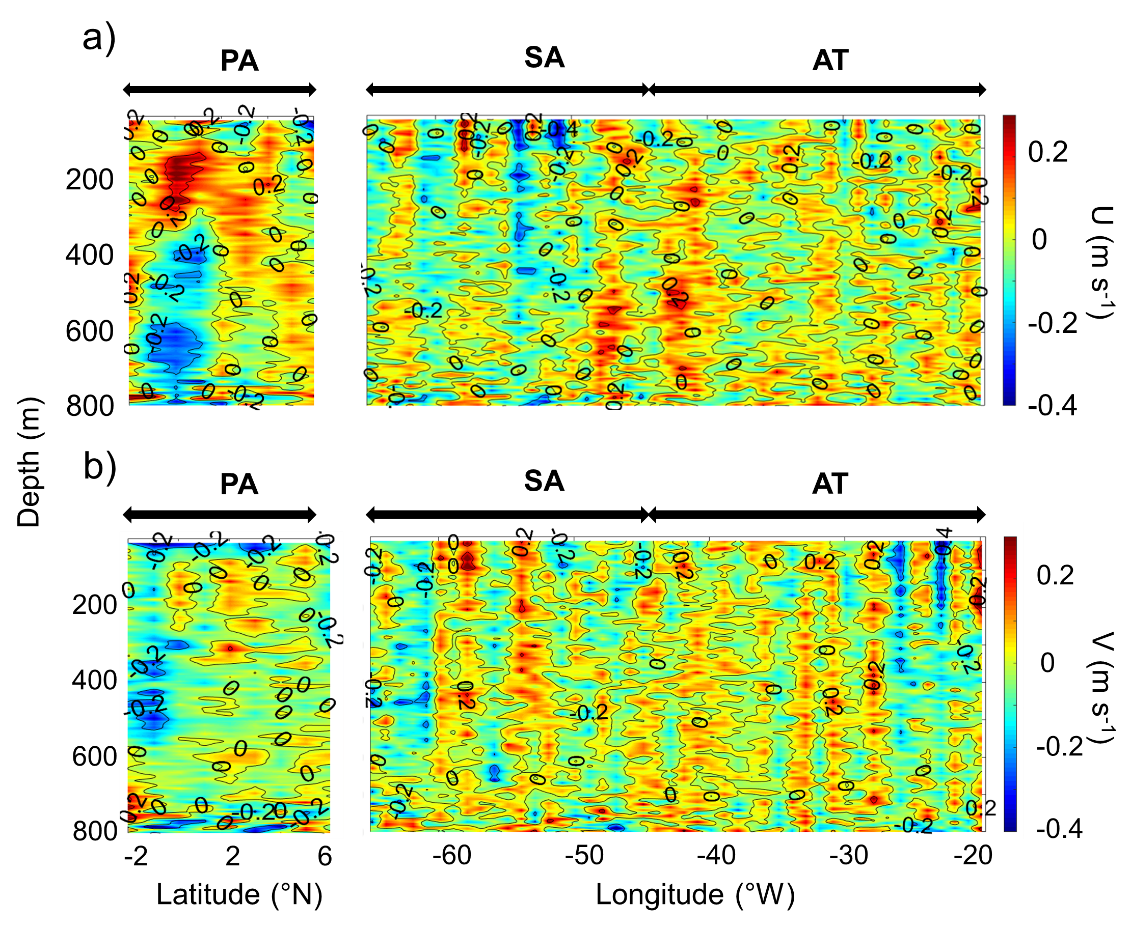


**Supplementary Fig. S8:** Ocean current velocity from Acoustic Doppler Current Profiler (ADCP at 75 kHz) along the water column (0 to 800 m depth) of (a) eastward “U” and (b) northward “V” components velocities in the eastern tropical Atlantic Ocean (AT), Sargasso Sea (SA) and Eastern tropical Pacific Ocean (PA). Acoustic Doppler Current Profiler (ADCP, 75 kHz) velocity measurements showing (a) eastward (U) and (b) northward (V) components from 0-800 m depth across the eastern tropical Atlantic (AT), Sargasso Sea (SA), and eastern tropical Pacific (PA). Color scale represents velocity (m s⁻¹): red = positive direction (east/north), blue = negative direction (west/south).


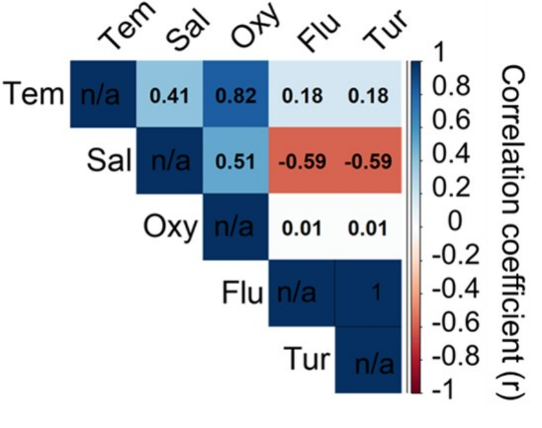


**Supplementary Fig. S9**: Results of correlation test for predictor variables: Sea temperature (Tem), Salinity (Sal), dissolved oxygen (Oxy), fluorescence (Flu) and water turbidity (Tur).


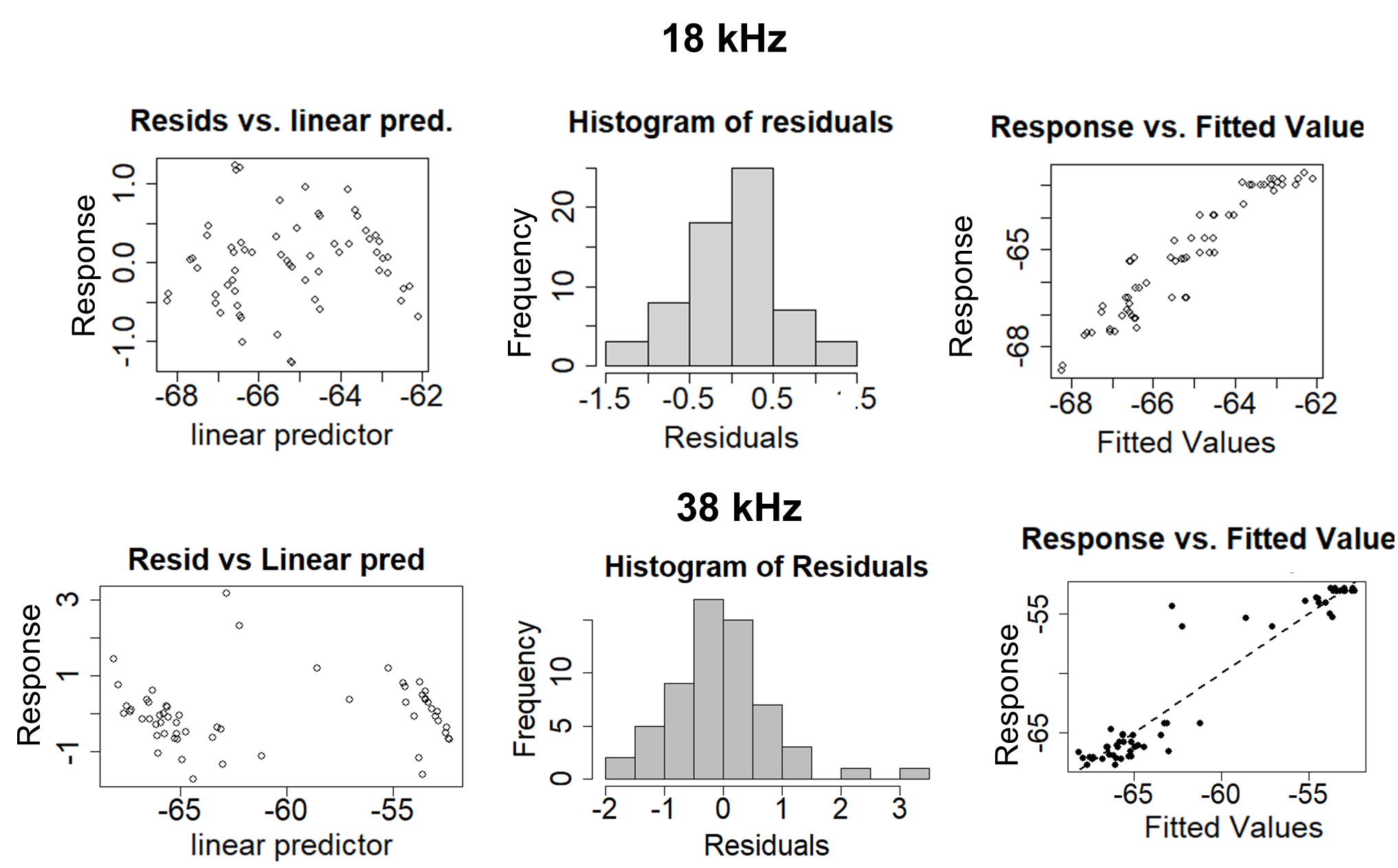


**Supplementary Fig. S10**: Diagnostic plots of the fitted GAM models between the mean volume backscattering strength (S_v_, in dB) of SSLs, a proxy of relative micronektonic density, and environmental parameters at 18 (Top) and 38 kHz (Down)

**Supplementary Table S1:** Analysis of variance (ANOVA) to test for statistically significant differences in the acoustic data between the locations (before and beyond Atlantic shear). (a) The ANOVA results (F-statistic = 11789, p-value < 2e-16) indicated a highly significant difference in the acoustic backscatter between the two locations. (b) Tukey's Honest Significant Difference (HSD) test revealed that the mean acoustic backscatter value beyond the shear location was significantly lower than before the shear, with a 95% confidence interval for the mean difference ranging from -1.37 to -1.32.

a)

Summary (anova_result)

|  | Df | Sum Sq | Mean Sq | F value | Pr(>F) |
| --- | --- | --- | --- | --- | --- |
| Locations | 1 | 1153783 | 1153783 | 11789 | <2e-16 *** |
| Residuals | 2544256 | 249010828 | 98 |  |  |

b)

TukeyHSD (anova_result)

|  | Difference | lwr | upr | Adjusted p-value |
| --- | --- | --- | --- | --- |
| Locations | -1.346826 | -1.371138 | -1.322513 | 0 |

**The Caribbean Sea**

Along the SO287 Connect sea survey, we crossed the Caribbean Sea, but no CTD stations were performed. Thus, we have excluded this data from the modelling exercise and analysis in relation to oceanographic features. Nevertheless, the echosounder (38 kHz) has provided interesting records and added new information about the variation of the pelagic landscape along the survey track. The SO287 Connect cruise was conducted on board the research vessel R/V SONNE along a transect (Supplementary Fig. 9a) from the Canary Islands to Ecuador, crossing the Northeast Atlantic, the Sargasso Sea, and the Caribbean Sea before entering the Eastern Tropical Pacific Ocean. The Caribbean Sea is influenced by oceanographic processes, including coastal upwelling and energetic mesoscale eddies (Torres et al., 2023). The Eastern Tropical Pacific, characterised by equatorial upwelling and El Niño/La Niña events, exhibits Complex Ocean dynamics (Kessler, 2006). We found that the clustering of SSL acoustic data resulted in three clusters spatially distributed across the surveyed area (Supplementary Fig. 9). Adding the Caribbean Sea (CA), four regions with different SSL features emerged: the Eastern Tropical North Atlantic Ocean (AT), the Sargasso Sea (SA), the Caribbean Sea (CA), and the Eastern Tropical Pacific Ocean (PA).

The SSLs in the CA exhibited characteristics similar to those of the SA but with a higher acoustic density. Considering the variability of oceanographic parameters per clustered region already reported for AT, SA, and PA, in the SA and the Northern part of the Caribbean, the thermocline deepens to 300 m, while in the AT and south CA regions, it typically ranges between 90 and 110 m. The PA and south CA regions exhibited the highest chlorophyll-a concentrations, ranging from 0.4 to 1.5 µg m^-3^, particularly at the surface (depth < 100 m). The epipelagic layer remains consistently stable across all regions, while the deep layer exhibits a descent from the end of the AT to the SA and then rises toward the CA.

**Additional discussion *vs* Caribbean Sea (CA)**

The AT and PA regions exhibited the thickest and longest SSLs, indicating higher concentrations of acoustic scatterers in these region *vs.* CA and SA. The SSLs in the CA displayed traits similar to those of SA, such as being deep, short, and thin, with the notable distinction of a higher acoustic density. This difference could be attributed to variations in the composition of acoustic scatterers in SSLs within the CA region compared to SA, as well as potential influences from local oceanographic features. The CA is significantly influenced by various oceanographic processes, including upwelling and large mesoscale eddies (e.g., Torres et al., 2023; Jouanno and Sheinbaum, 2013). Early investigations in the (CA) reported upwelling and increased primary production along the coast of Venezuela (Richards, 1960) and the Guajira Peninsula in Colombia (Curl, 1960). These processes are crucial in transporting nutrient-rich waters from the deeper ocean layers to the surface. Estimates indicate that around 95% of the small pelagic biomass within the CA relies on the enhanced biological productivity driven by the upwelling waters originating from the South Caribbean Upwelling System (Rueda-Roa and Muller-Karger, 2013). The increased primary productivity resulting from the nutrient influx (Muller-Karger et al., 2001) sustains the growth of zooplankton and micronekton organisms, thereby contributing to the high relative biomass of SSLs in the CA region.


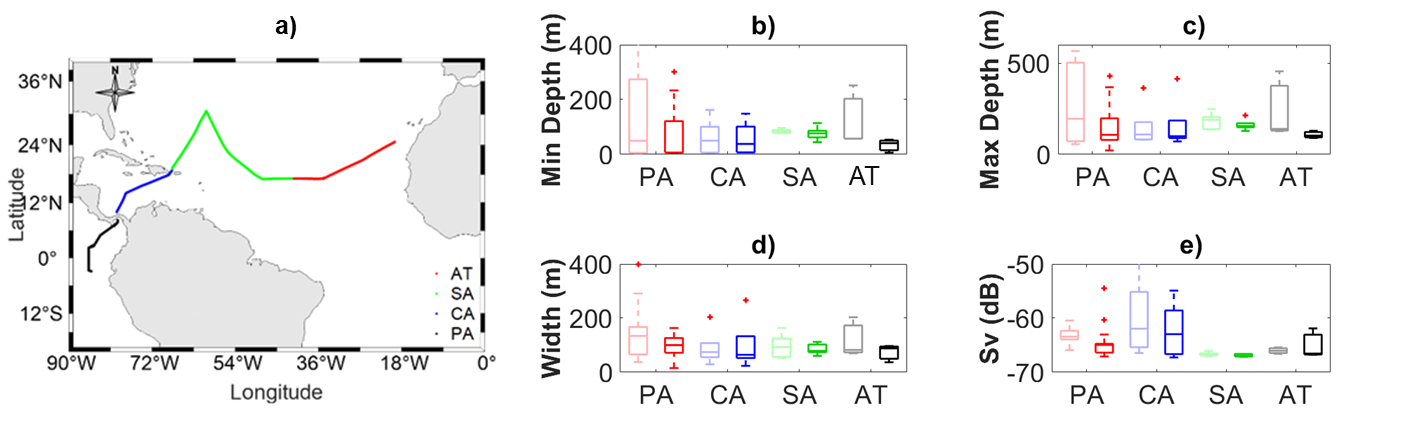


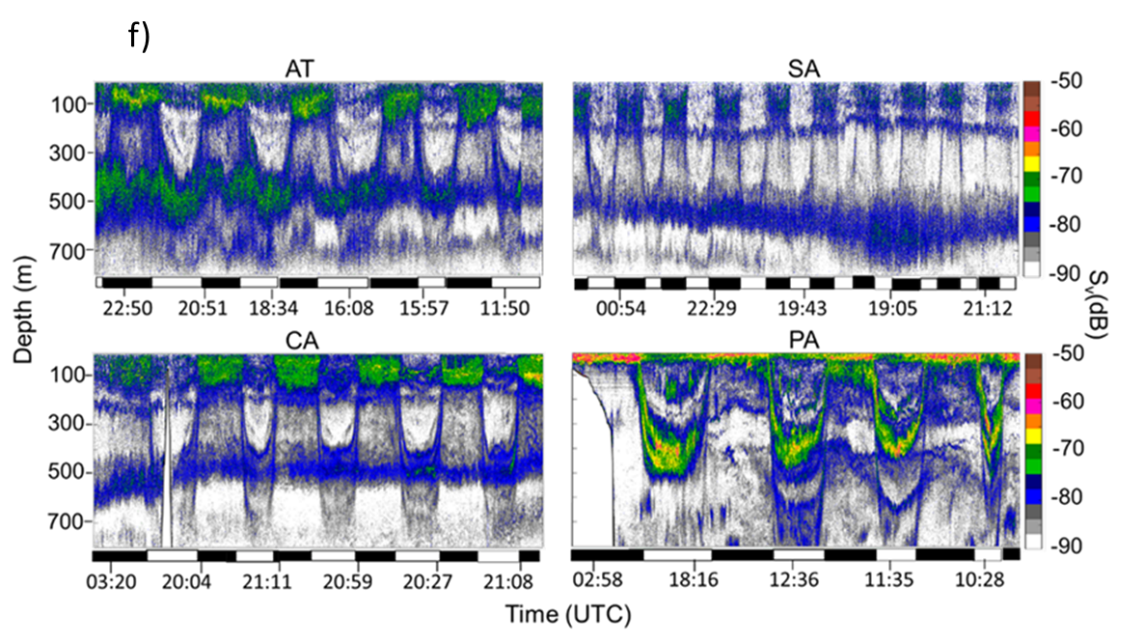


***Supplementary Fig.* S11**: k-means clustering of (18 kHz, light colors and 38 kHz, bold colors) acoustic data allows discrimination of four areas from 20°W to 90°W (Fig. 1). a) Map of the survey track with each day coloured by its resulting cluster: Eastern Tropical Pacific Ocean (red; PA), the Caribbean Sea (blue; CA); Sargasso Sea (green; SA), and Eastern Tropical North Atlantic Ocean (black; AT). b-e) Boxplots (minimum, maximum, median, and outlier) of Sound Scattering Layer (SSL) metrics for each identified cluster: b) SSL minimal depth (m), c) maximal depth (m), d) width (m), and e) mean volume backscattering strength S_v_ (dB) proxy of micronektonic acoustic backscatter. f) Echograms (Echointegration at -100 dB detection from 38 kHz echosounder) showing micronektonic diel vertical migration: in Eastern Tropical North Atlantic Ocean (AT), Sargasso Sea (SA), Caribbean Sea (CA) and Eastern Tropical Pacific Ocean (PA). The bottom axis depicts the diel period: day (white) and night (black) (adapted from Figure 4).
